# Urodynamic Investigation: A Valid Tool to Define Normal Lower Urinary Tract Function?

**DOI:** 10.1371/journal.pone.0163847

**Published:** 2016-10-13

**Authors:** Lorenz Leitner, Matthias Walter, Ulla Sammer, Stephanie C. Knüpfer, Ulrich Mehnert, Thomas M. Kessler

**Affiliations:** 1 Neuro-Urology, Spinal Cord Injury Center & Research, University of Zürich, Balgrist University Hospital, Zürich, Switzerland; 2 Department of Urology, University Hospital Basel, Basel, Switzerland; Carolina Urologic Research Center, UNITED STATES

## Abstract

**Objectives:**

To evaluate whether urodynamic investigation (UDI), the gold standard to assess refractory lower urinary tract symptoms (LUTS), is appropriate to select healthy volunteers with apparent normal lower urinary tract function as control subjects for comparative studies.

**Subjects and Methods:**

42 healthy subjects (22 women, mean age 32±10 years; 20 men, mean age 37±12 years) without LUTS were included into this prospective single-centre cohort study. All subjects recorded a 3-day bladder diary, completed validated questionnaires regarding LUTS, and underwent neuro-urological assessment as well as free uroflowmetry. Same session repeat UDI was performed according to “Good Urodynamic Practice” recommended by the International Continence Society, but using an air-charged instead of a water-filled catheter, and evaluated by a blinded investigator.

**Results:**

All 3-day bladder diaries, LUTS questionnaires, neuro-urological assessments and free uroflowmetries were within normal limits. Overall (either during the first or second UDI), same session repeat UDI revealed pathological findings in 71% (30/42): Detrusor overactivity was detected in 14% (3/22) and 30% (6/20), post void residual >100mL in 14% (3/22) and 25% (5/20), bladder outlet obstruction in 9% (2/22) and 20% (4/20) and detrusor sphincter dyssynergia in 77% (17/22) and 65% (13/20) of our women and men, respectively.

Repeatability of detrusor overactivity (κ = 0.78, 95% CI: 0.54–1.02) and detrusor sphincter dyssynergia (κ = 0.77, 95% CI: 0.55–0.98) showed substantial agreement between both UDIs. All other assessed urodynamic parameters had wide 95% limits of agreement for differences in the parameters indicating poor repeatability.

**Conclusions:**

More than 70% of our healthy subjects showed pathological urodynamic findings. Although UDI is the gold standard to assess refractory LUTS, it seems not to be applicable in healthy subjects to define normal lower urinary tract function. Therefore, we do not recommend using UDI to select healthy control subjects.

## Introduction

Urodynamic investigation (UDI) is the gold standard to assess refractory lower urinary tract symptoms (LUTS), i.e. to detect lower urinary tract dysfunction (LUTD) [1]. However, patients with LUTS do not necessarily show pathological urodynamic findings [2]. In general, knowledge of what constitutes a normal organ function is based on investigation of healthy subjects. However, previous UDIs in symptom-free healthy subjects showed a wide range of urodynamic findings including pathological results [3–6]. Thus, the value of UDIs in asymptomatic subjects, i.e. subjects without LUTS, is largely unknown.

The aim of the present study was to assess whether UDI (filling cystometry and pressure-flow study (PFS)), is appropriate to select healthy volunteers, with apparent normal lower urinary tract function, as control subjects for comparative studies with patients suffering from LUTD.

## Subjects and Methods

### Ethics

This prospective cohort study has been approved by the local ethics committee (Kantonale Ethikkommission Zürich, KEK-ZH-Nr. 2011–0346), is registered at ClinicalTrials.gov (NCT01768910) and was performed at a single university spinal cord injury centre.

### Subjects

**Fig 1:**
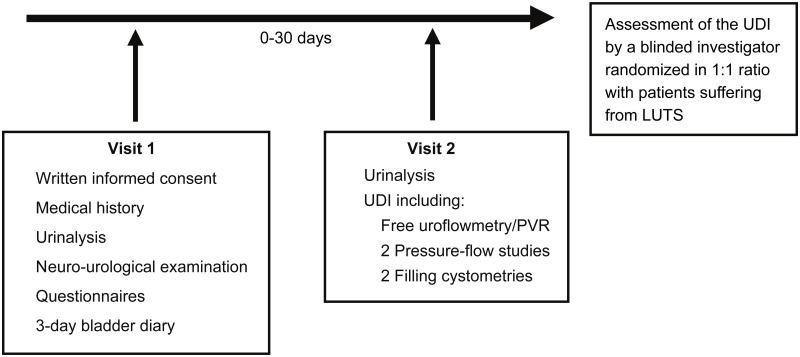
Flow chart of all visits. On subjects’ request UDI could be performed at the first visit, if the 3-day bladder diary was already available and all further inclusion criteria were met. LUTS = Lower urinary tract symptoms, PVR = Post void residual, UDI = Urodynamic investigation.

Healthy subjects (22 women, mean age 32±10 years; 20 men, mean age 37±12 years) were recruited by public advertisement. Inclusion criteria were: (1) age between 18 and 55 years, (2) no LUTS, (3) no history of previous lower urinary tract surgery, (4) no history of previous or current neurological diseases, including diabetic neuropathy, and (5) no current medication. The flowchart of all visits can be found in Fig 1. During screening visit, healthy subjects were informed about the study details, i.e. aims, methods, possible risks, and side effects. After obtaining written informed consent, the following data was acquired: 3-day bladder diary and validated, standardised questionnaires (in German language) regarding LUTS, quality of life (QoL), mental status and depression, i.e. International Consultation on Incontinence Modular Questionnaire modules (ICIQ-FUTS, ICIQ-MLUTS) [7], the Overactive Bladder Questionnaire short-form (OAB-q SF) [8], the Mini Mental Status Examination [9], and the Hospital Anxiety and Depression Scale [10]. The scores for ICIQ-FLUTS were derived according to the publication by Brookes et al. [11] and subdivided in filling, voiding, and incontinence symptoms. For ICIQ-MLUTS, the recommendation by the ICS was followed subdividing the scores in voiding and incontinence symptoms [12]. Neuro-urological assessment [13] included medical history, examination of urogenital sensation, bulbocavernosus reflex (performed by squeezing the clitoris or glans during digito-rectal examination and pelvic floor electromyography (EMG)), anal reflex, anal sphincter tone, and anal squeeze response. Free uroflowmetry was performed (uroflowmetry has been repeated in case of voided volume <200 mL) and post void residual (PVR) was measured by ultrasound. Prior to invasive UDI, urinary tract infection (UTI) and pregnancy have been excluded. Subjects were instructed to reduce their fluid intake two hours before the UDI. As a requirement of the local ethics committee, healthy subjects obtained a minimal financial compensation.

### Urodynamic investigation

UDIs were performed by two examiners (LL and MW) and comprised same session repeat filling cystometry and PFS. UDI was performed according to “Good Urodynamic Practice” recommended by the ICS [1] but using an air-charged instead of a water-filled catheter. No minimal amplitude threshold to define detrusor overactivity was set. Bladder outlet obstruction was defined according to Defreitas et al. [14] as maximum flow rate (Qmax) <12mL/s and detrusor pressure (pDet) Qmax >25cmH_2_O or according to the Abrams-Griffiths nomogram [15] for women and men, respectively. According to the recommendation of the ICS [16] involuntary contraction of the urethral and/or periurethral striated muscle, i.e. elevated EMG signal, during detrusor contraction, was defined as detrusor sphincter dyssynergia.

An air-charged, three-way 7 Fr transurethral catheter (T-DOC-7FD, Laborie Medical Technologies, Ontario, Canada) and a common rectal catheter for simultaneous measurements of vesical and abdominal pressure were used. Both catheters were attached to the body surface, using tape to assure correct placement during the entire UDI. The vesical catheter was attached close to the urethral meatus to avoid expelling during UDI. Surface electrodes (Neotrode II, ConMed Cooperation, New York, USA) were placed bilaterally around the external anal sphincter to record activity of the pelvic floor, i.e. EMG. For comparison reasons between women and men, free uroflowmetry, filling cystometry, and PFS were performed in sitting position in all subjects, even if standing was the indicated preferred voiding position in some male volunteers. During invasive UDI intravesical pressure, intraabdominal pressure, pDet, pelvic floor EMG as well as urinary flow (Q), and voided volume in the voiding phase, using Laborie Goby and Laborie Urocap IV system (Laborie Medical Technologies, Ontario, Canada), were continuously recorded. Prior to each measurement, proper placement and function of catheters and electrodes were assessed by cough, pelvic floor contraction and elicitation of the bulbocavernosus reflex. In addition, subjects were asked to cough during cystometry, i.e. after every 100 mL, to verify correct placement and function of catheters. Moreover, plausibility and quality control of clinical and urodynamic data was performed: Healthy volunteers reported their sensation during investigations and free uroflowmetry and flow patterns during PFS has been compared.

Continuous bladder filling was carried out using body warm (37°C) sterile saline at a filling speed of 30 mL per minute for filling cystometry. Subjects were asked to report the following sensations: first sensation of bladder filling (FSF), first desire to void (FDV) and strong desire to void (SDV) during bladder filling when perceived, the infused volume at each time point was acquired and correction for permanent urine self-production was made for maximum cystometric capacity (MCC). Permission to void was given, when SDV was reported and PFS was performed in privacy (i.e. no investigator in the room). After PFS, PVR was measured following bladder emptying by a single use 10 Fr transurethral catheter (LoFric Origo/Sense, Wellspect HealthCare, Mölndal, Sweden). MCC was defined as the sum of voided volume and PVR. If no micturition could be initiated, the whole catheterised volume was classified as PVR. Subjects were blinded to UDI parameters and findings.

UDIs were assessed by a blinded investigator (US), i.e. an experienced consultant in neuro-urology. The investigator had no knowledge of subjects’ history and clinical examination. Gender only was unmasked to address the different definitions of bladder outlet obstruction. UDIs were presented to the investigator in a randomised order mixed with patients suffering from LUTS in a 1:1 ratio.

### Statistical analysis

Data distribution was tested by Q-Q plots. Approximately normally distributed data (FSF, FDV, SDV, MCC, compliance, pDet max during voiding, pDet Qmax, and voided volume) were presented as mean ± standard deviation (SD), skewed data (pDet max during storage and PVR) as median and interquartile range. Comparing unrelated samples, i.e. women vs. men, the unpaired t test was used for approximately normally distributed data and the Mann-Whitney U test for skewed data, respectively. For comparison of unrelated binary data Fisher’s exact test was used.

To compare the difference between quantitative parameters between the first and second UDI, i.e. repeatability, the generally accepted Bland and Altman 95% limits of agreement [17] were applied (and therefore no p-values are given). The κ statistic was used to investigate agreement of the presence or absence of detrusor overactivity and detrusor sphincter dyssynergia between both UDIs.

Statistical analyses were performed using IBM's Statistical Package for the Social Sciences (SPSS) V22 (IBM SPSS Statistics for Windows, Version 22.0. Armonk, NY, USA) with p<0.05 considered statistically significant.

## Results

### Baseline characteristics

Baseline characteristics of the 42 subjects (22 women and 20 men, all Caucasians) are shown in Table 1. Medical history, 3-day bladder diaries, questionnaires regarding LUTS and QoL, neuro-urological assessment, free uroflowmetry, PVR, and urine samples, were without pathological findings in all subjects.

**Table 1:** Baseline characteristics.

Baseline characteristics	Women	Men	p Value
	(n = 22)	(n = 20)	
*Age [year]*	32 ± 10	37 ± 12	0.13^†^
* 18–39 years*	15/22 (68%)	12/20 (60%)	
* 40–55 years*	7/22 (32%)	8/20 (40%)	
*Weight [kg]*	61 ± 7	76 ± 6	<0.01**^/^^†^
*3-day bladder diary*			
Micturition frequency per 24 hours	5.7 ± 1.2	5.9 ± 1.2	0.44^†^
Micturition volume per micturition [mL]	305 ± 110	305 ± 65	0.95^†^
Fluid intake per 24 hours [mL]	1920 ± 790	2230 ± 775	0.21^†^
*Questionnaires*			
ICIQ-FLUTS/MLUTS***			
Filling symptoms	1.1 ± 0.9	-	
Voiding symptoms	0.2 ± 0.4	3 ± 2.8	
Incontinence symptoms	0.3 ± 0.8	1.4 ± 2.5	
OAB-q SF			
Symptoms	6.9 ± 1.2	7.6 ± 1.6	0.18^†^
QoL	14.1 ± 0.9	13.9 ± 0.9	0.64^†^
HADS			
Anxiety	2.8 ± 2.2	1.4 ± 1.5	0.01**^/^^†^
Depression	1 ± 1.5	1.1 ± 1.5	0.92^†^
MMSE	29.6 ± 0.5	29.7 ± 0.6	0.45^†^
*Neuro-urological examination*			
Urogenital sensation (intact/impaired)	22/0	20/0	
Bulbocavernosus reflex (intact/impaired)	22/0	20/0	
Anal reflex (intact/impaired)	22/0	20/0	
Anal sphincter tone (intact/impaired)	22/0	22/0	
Anal squeeze response (intact/impaired)	22/0	20/0	
*Free uroflowmetry (Voided volume > 200 mL)*			
Maximum flow rate [mL/s]	29 ± 11	25 ± 6	0.12^†^
Post void residual [mL]*	0 (0–0)	0 (0–0)	0.18^††^

* = parameter with a skewed distribution (presented as median and interquartile range); all other parameters are approximately normally distributed (presented as mean ± standard deviation)

** = significant difference between gender using an unpaired t test

*** = due to the different scoring systems, female and male subjects have not been compared

^†^ = unpaired t test

^††^ = Mann-Whitney U test

ICIQ = International Consultation on Incontinence Modular Questionnaire, FLUTS = Female lower urinary tract symptoms, MLUTS = Male lower urinary tract symptoms, OAB-q SF = The Overactive Bladder Questionnaire short-form, QoL = Quality of life, HADS = Hospital Anxiety and Depression Scale, MMSE = Mini-Mental State Examination.

### Same session repeat urodynamic investigations

**Fig 2:**
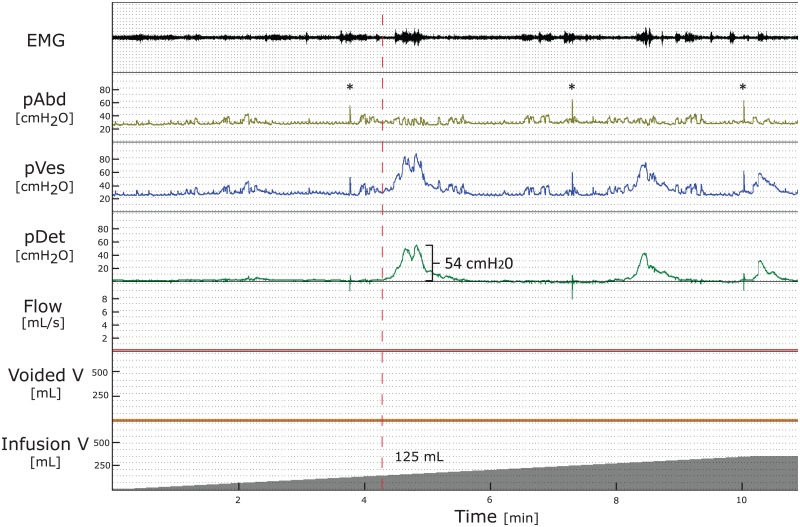
Pathological findings during filling cystometry. Filling cystometry (bladder filling with 30 mL/min) of a 51 years old healthy man: First detrusor overactivity occurs at 125 mL (red-dashed line) with maximum pDet of 54 cmH2O but no detrusor overactivity incontinence, the maximum cystometric capacity is 345 mL. The associated pressure flow study is normal and not shown in the figure.

**Fig 3:**
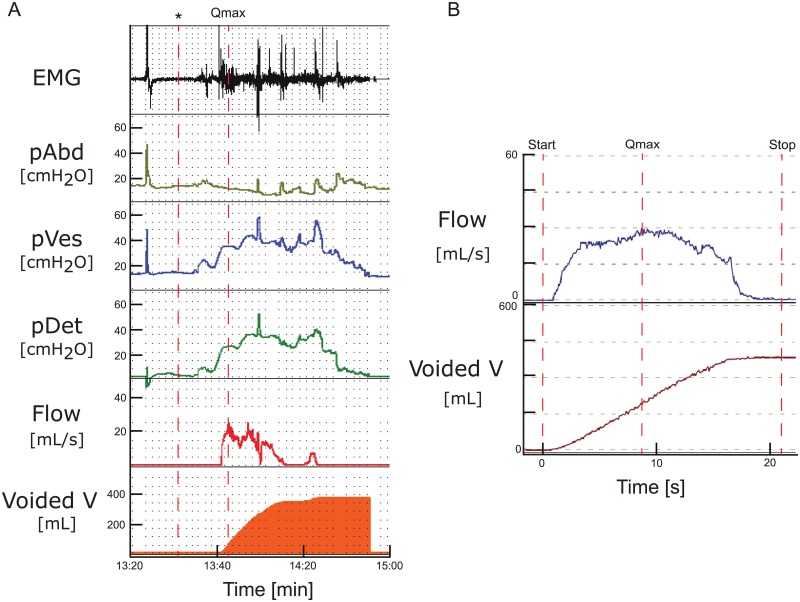
Pathological findings during pressure flow study. a) Pressure flow study of a 24 years old healthy woman: The first spike indicates a cough to evaluate correct catheter placement, thereafter permission to void (*) is obtained. The EMG signal during pressure flow study is elevated, the flow is interrupted; consequent spikes in the vesical and detrusor pressure can be seen. Maximum cystometric capacity/voided volume 395 mL, maximum pDet voiding 31 cmH2O at maximum flow rate 25 mL/s, no post void residual. b) The free uroflowmetry in the same subject reveals a normal flow-curve. Maximum flow rate 30 mL/s, voided volume 390 mL, no post void residual. In line with the International Continence Society, we interpret detrusor sphincter dyssynergia in this pressure flow study as a phenomenon provoked by the examination itself, especially also considering the normal flow pattern and the lack of PVR during free uroflowmetry. * = permission to void, EMG = electromyography, pAbd = intraabdominal pressure, pVes = intravesical pressure, pDet = detrusor pressure, Qmax = maximum flow rate, V = volume.

Urodynamic findings are shown in Table 2. Significant differences were found between genders for volumes at FSF, pDet Qmax and Qmax within first and second UDI as well as for maximum pDet during the first PFS within first UDI. Overall, UDI revealed pathological findings (Table 3, Figs 2 and 3) in 71% (30/42): Detrusor overactivity (p = 0.18) was detected in 14% (3/22, 1 reported synchronous urgency) and 30% (6/20, 3 reported synchronous urgency), bladder outlet obstruction (p = 0.29) in 9% (2/22) and 20% (4/20), detrusor sphincter dyssynergia (p = 0.3) in 77% (17/22) and 65% (13/20), and PVR >100mL (p = 0.29) in 14% (3/22) and 25% (5/20) of our women and men, respectively. Pathological findings were similar during both UDIs (p>0.05).

**Table 2:** Urodynamic findings.

	First investigation			Second investigation		
Urodynamic parameter	Women	Men	p Value	Women	Men	p Value
**Filling cystometry**						
FSF [mL]	75 ± 75	130 ± 85	0.04**^/^^†^	70 ± 85	145 ± 95	0.01**^/^^†^
FDV [mL]	235 ± 110	215 ± 105	0.53^†^	265 ± 115	235 ± 145	0.49^†^
SDV [mL]	465 ± 155	435 ± 140	0.49^†^	530 ± 165	450 ± 150	0.11^†^
MCC [mL]	560 ± 185	500 ± 165	0.27^†^	550 ± 160	500 ± 160	0.33^†^
Compliance [mL/cmH2O]	268 ± 204	241 ± 183	0.66^†^	300 ± 211	193 ± 195	0.1^†^
pDet max during storage [cmH2O]*	4 (3–7)	6 (2–12)	0.46^††^	5 (2–8)	6 (3–11)	0.26^††^
**Pressure flow study**						
pDet max during voiding [cmH2O]	46 ± 28	65 ± 25	0.03**^/^^†^	48 ± 24	62 ± 33	0.11^†^
pDet Qmax [cmH2O]	29 ± 11	51 ± 16	<0.01**^/^^†^	32 ± 16	47 ± 20	<0.01**^/^^†^
Qmax [mL/s]	29 ± 17	19 ± 8	0.02**^/^^†^	28 ± 12	20 ± 8	0.03**^/^^†^
Voided volume [mL]	510 ± 255	445 ± 190	0.36^†^	505 ± 185	450 ± 195	0.36^†^
PVR [mL]*	0 (0–15)	15 (0–105)	0.14^††^	0 (0–5)	0 (0–60)	0.21^††^

* = parameter with a skewed distribution (presented as median and interquartile range); all other parameters are approximately normally distributed (presented as mean ± standard deviation)

** = significant difference between gender using an unpaired t test

^†^ = unpaired t test

^††^ = Mann-Whitney U test

FSF = First sensation of filling, FDV = First desire to void, SDV = Strong desire to void, MCC = Maximum cystometric capacity, pDet max = Maximum detrusor pressure, pDet Qmax = Detrusor pressure at maximum flow rate, Qmax = Maximum flow rate, PVR = Post void residual.

**Table 3:** Pathological findings during urodynamic investigations.

	First investigation		Second investigation	
Urodynamic parameter	Women	Men	Women	Men
**Filling cystometry**				
Detrusor overactivity	14% (3/22)	30% (6/20)	14% (3/22)	25% (5/20)
Detrusor overactivity incontinence	14% (3/22)	5% (1/20)	0	0
Compliance < 20 [mL/cmH2O]	5% (1/22)	0	0	0
**Pressure flow study**				
Micturition not possible	9% (2/22)	5% (1/20)	9% (2/22)	5% (1/20)
Obstructed*	9% (2/22)	10% (2/20)	9% (2/22)	20% (4/20)
Equivocal*	-	20% (4/20)	-	5% (1/20)
Detrusor sphincter dyssynergia	77% (17/22)	65% (13/20)	77% (17/22)	65% (13/20)
PVR >100 mL	14% (3/22)	25% (5/20)	5% (1/22)	20% (4/20)

No significant difference for appearance of pathological findings between gender was seen, using Fisher’s exact test for comparison of unrelated binary data

Only one male patient showed detrusor overactivity during the first UDI but not during the second UDI, detrusor sphincter dyssynergia occurred each time in the same patient. One female subject and one male subject could not void during both, the first and the second UDI. Two other female subjects could either void during the first or second UDI, respectively. One female subject showed bladder outlet obstruction in PFS during both UDIs. Two other female subjects showed bladder outlet obstruction in PFS either during the first or second UDI. The two male subjects with bladder outlet obstruction in PFS during the first UDI showed also bladder outlet obstruction during the second UDI. The two male subjects with bladder outlet obstruction during the second UDI only were equivocal during the first UDI.

*according to Defreitas et al. and the Abrams-Griffiths nomogram for women and men, respectively (due to the different definitions, female and male subjects have not been compared)

PVR = Post void residual, PFS = Pressure flow study, UDI = Urodynamic investigation.

**Fig 4:**
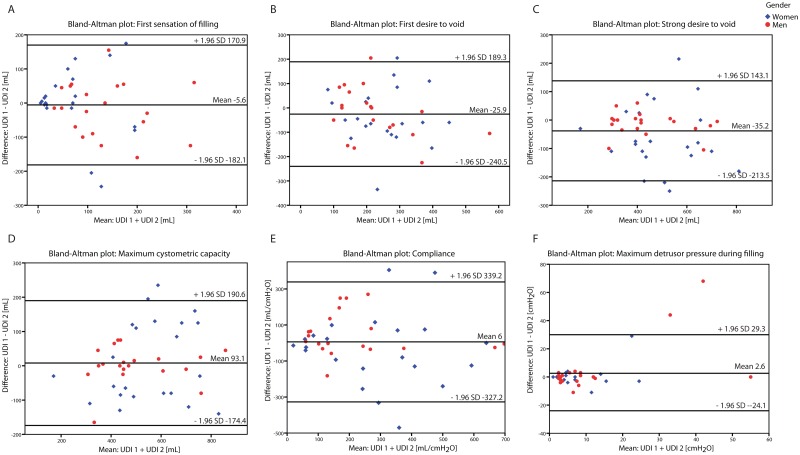
Difference against mean plot filling cystometry. Difference against mean plot for a) first sensation of filling, b) first desire to void, c) strong desire to void, d) maximum cystometric capacity, e) compliance, and f) maximum detrusor pressure during filling for UDI 1 vs. 2. Wide 95% limits of agreement reflect poor repeatability with unacceptable discrepancies between same session repeat UDIs. UDI = Urodynamic investigation.

**Fig 5:**
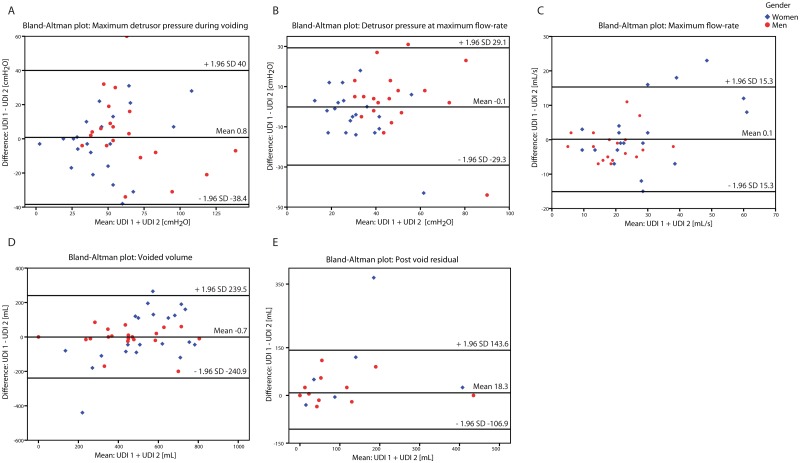
Difference against mean plot pressure flow study. Difference against mean plot for a) maximum detrusor pressure during voiding, b) detrusor pressure at maximum flow-rate, c) maximum flow rate, d) voided volume, and e) post void residual for UDI 1 vs. 2. Wide 95% limits of agreement reflect poor repeatability with unacceptable discrepancies between same session repeat UDIs. UDI = Urodynamic investigation.

Using the Bland and Altman method, there were wide 95% limits of agreement for differences in same session UDI parameters indicating poor repeatability (Figs 4 and 5).

Detrusor overactivity and detrusor sphincter dyssynergia were found in 9 (21%) versus 8 (19%) and in 30 (71%) versus 30 (71%) of the 42 subjects in UDI 1 versus 2, respectively. The repeatability of detecting detrusor overactivity (κ = 0.78, 95% CI 0.54–1.02) and detrusor sphincter dyssynergia (κ = 0.77, 95% CI 0.55–0.98) showed substantial agreement between both UDIs.

### Adverse events

An adverse event, as defined by the International Conference on Harmonisation (ICH) Good Clinical Practice (GCP) Guidelines (E6) [18] and International Organization for Standardization (ISO, 14155) [19], did not occur.

## Discussion

### Main findings

In more than 70% of our healthy subjects UDI revealed pathological findings, most commonly detrusor sphincter dyssynergia defined as a detrusor contraction concurrent with an involuntary contraction of the urethral and/or periurethral striated muscle [16]. Although UDI is the gold standard to assess refractory LUTS, it seems not to be applicable in apparently healthy subjects to define normal lower urinary tract function. Therefore, we do not recommend using UDI to select healthy subjects as controls for comparative studies.

### Findings in the context of existing evidence

The literature on UDI findings in healthy subjects is very limited. In line with previous studies [5, 6, 20], we found significantly lower bladder volumes at FSF in women compared to men. In contrast to other studies, the volumes at FSF were relatively low, what could be instruction related, e.g. filling sensation versus awareness of the urethral catheter irritating the bladder wall, as subjects were instructed to report the first bladder perception during filling cystometry. However, as FSF is a very subjective sensation wide variability seems plausible. We also detected significantly lower pDet during PFS and a higher Qmax in women compared to men, i.e. phenomena well known from UDI in patients with LUTS [14, 15]. In healthy volunteers, involuntary detrusor contractions would be expected, but earlier studies revealed detrusor overactivity in 4–18% during conventional and up to 69% during ambulatory urodynamics in asymptomatic subjects [5, 21, 22]. It could be shown that the method of instruction, i.e. neither try to void nor to inhibit micturition during bladder filling versus simply report sensation to the examiner, affects the incidence of involuntary detrusor contractions [23]. Overall, detrusor overactivity was detected in 21% of our healthy subjects. It was found more frequently in men (30%) than in women (14%). The reason for this finding is unclear but anatomical gender differences may be relevant as the catheter could irritate the urethral mucosa causing detrusor overactivity during UDI. Lower bladder capacity in bladder diary compared to UDI might be explained by the fact that in everyday life the bladder is also emptied on occasion and not only at SDV. Moreover, bladder volume effects and the non-physiological high filling rate (30 mL/s) cannot be ruled out completely as an underlying cause for detrusor overactivity episodes.

Regarding parameters from the PFS, our values are in accordance with the very limited available literature [3, 21].

In our cohort, detrusor sphincter dyssynergia was the most commonly detected pathological finding. It might be argued, that detrusor sphincter dyscoordination or dysfunctional voiding would be more appropriate terms than detrusor sphincter dyssynergia to describe our findings. However, according to ICS terminology, detrusor sphincter dyssynergia is a pure urodynamic diagnosis defined as detrusor contraction concurrent with an involuntary contraction of the urethral and/or periurethral striated muscle and typically occurs in patients with a supra-sacral lesion [16], but an underlying neurological disorder has not been a prerequisite for the definition. Indeed, a percentage of 71% (30/42) undetected underlying neurological disorders in presumably healthy volunteers is very unlikely. Considering the normal flow pattern and lack of PVR during free uroflowmetry, the high rate of detrusor sphincter dyssynergia is most likely a phenomenon provoked by the examination itself, i.e. stiff irrigating catheter and the unselective recording of the surface EMG. Although detrusor sphincter dyscoordination is commonly used in German speaking countries for detrusor sphincter dyssynergia in neurologically normal individuals, it is not according to the ICS terminology. Moreover, dysfunctional voiding is not a very specific term defined as intermittent and/or fluctuating flow rate due to involuntary intermittent contractions of the periurethral striated muscle during voiding [16]. Importantly, detrusor contraction is not considered by this definition.

Combined pelvic floor EMG and videocystourethrography during UDI are the most accepted and widely agreed methods for diagnosing detrusor sphincter dyssynergia [24]. In the present study, however, we refrained from using videocystourethrography to avoid radiation exposure to our healthy volunteers. Therefore, we only used pelvic floor EMG to record detrusor sphincter dyssynergia. In line with our findings, an increased EMG signal during voiding has been reported in more than half of 321 neurologically normal female patients with predominant stress urinary incontinence [25], not demonstrating the typically expected relaxation during normal voiding. To distinguish between a solitary EMG activation of the intraurethral striated sphincter and a general muscle activation of the pelvic floor, an intramural sphincter needle electrode EMG would have been needed. However, surface electrodes are used in daily clinical practice and placing needle electrodes in healthy subjects would cause ethical concerns.

Repeatability of detrusor overactivity and detrusor sphincter dyssynergia showed substantial agreement between both UDIs. For all other urodynamic parameters assessed, however, there were wide 95% limits of agreement for differences in the parameters, indicating poor repeatability. These findings are very similar to the results in patients with neurogenic LUTD [24, 26] and highlight the importance of same session repeat UDI for clinical decision making.

### Implications for practice

The principal aim of a UDI is to detect and specify LUTD related to the patients’ symptoms helping to select the most appropriate treatment. However, Katz et al. [2] reported discordance between clinical and UDI findings in 45% of patients with storage, 25% with voiding and 54% with combined storage and voiding problems, respectively. Although UDI did change clinical decision making and therapy in a relevant percentage of men with voiding dysfunction, it remained unclear whether this also leads to reduced symptoms after treatment [27]. Based on our study, identifying pathological findings in more than 70%, UDI seems not be appropriate in healthy subjects to define normal lower urinary tract function. In patients complaining about LUTS, a urodynamic pattern such as in Fig 2 would lead to the initiation of a treatment. However, pathological urodynamic patterns may be iatrogenic, simply induced by the UDI itself and not by an underlying disease. UDI is always a non-physiological attempt to mimic storage and voiding phase and as such it is subject to many errors. In fact, in clinical practice we compare the Q shape of the free uroflowmetry with the PFS and if the free flow Q trace is normal we would disregard an eventual pathological flow pattern of the PFS and attribute it may be secondary to the test situation. Further, social inhibition can be a reason for patients not being able to void. According to the ICS, findings of PVR during invasive UDI should be considered as an artefact caused by UDI, if no PVR is demonstrated after free uroflowmetry [16]. If voiding symptoms are not the urodynamic question the UDI is being performed to answer, such findings may be irrelevant in daily clinical practice. Thus, for clinical decision making, it is crucial that UDI findings reflect the situation in daily life, i.e. the patients’ symptoms. Validated questionnaires and bladder diaries are of great value since they provide an objective patient-reported measure of LUTS [28].

### Implications for research

In contrast to patients with LUTS, there is no consensus or standardisation regarding normal UDI parameters and/or nomograms in healthy subjects. When applying generally agreed definitions [16], one will encounter a very high percentage of pathological findings. Therefore, we do not recommend using UDI to select healthy subjects as controls for comparative studies but rather rely on bladder diaries, validated questionnaires and neuro-urological assessment [13]. Detrusor sphincter dyssynergia was one of the predominant findings in our study. Intermittent involuntary contraction of the urethral and/or periurethral striated muscle due to a contact with the transurethral urodynamic catheter could have contributed to these findings. This may also be relevant in patients with preserved sensory function [29]. Comparison between free uroflowmetry and invasive UDI both conducted with pelvic floor EMG could give further insights into catheter-induced changes.

Thus, many important questions remain to be answered highly warranting further investigations in healthy subjects.

### Limitations of the study

Although to the best of our knowledge, this is the first prospective study to assess the value of UDI to appropriately select healthy volunteers with apparent normal lower urinary tract function as control subjects, some limitations should be addressed. Since we used an air-charged catheter for UDI, it is unclear whether the results can be extrapolated to UDI with a water-filled catheter, especially considering that for urethral pressure measurement urodynamic catheters cannot be used interchangeably because of insufficient agreement [30] and that this may also be the case for cystometry and PFS. The response to pressure changes and the frequency rate of attenuated signals are different between the air-charged and water-filled systems [31, 32]. Although there are no randomised controlled trials, higher pressures were consistently found with the air-charged compared with the water-filled catheters [32, 33]. The Abrams-Griffiths nomogram to define bladder outlet obstruction in men [15] has only been validated for water-filled systems, but lacking alternatives we applied it also in our study, so that bladder outlet obstruction may have been overestimated. Considering the relative young age of the studied cohort, a selection bias cannot be completely excluded. Based on age-related urodynamic changes [34], it may be hypothesised that older subjects are more vulnerable for UDI-induced pathological findings. Subsequently, an older population of healthy subjects could even show a higher percentage of pathological results. Moreover, we used a constant filling rate of 30 mL/min and did not explore a potential impact by changing the infusion speed between UDIs and/or subjects. Taking into account that a physiological filling rate (mL/min) has been defined as less than body weight in kg divided by 4 [16], we cannot exclude that a supra-physiological infusion speed might have provoked detrusor overactivity contributing to the high incidence of detrusor overactivity found in our healthy volunteers. In addition, different bladder volumes in free uroflowmetry and invasive UDI might be relevant and complicate comparability of these parameters.

## Conclusions

UDI is the gold standard to assess refractory LUTS, i.e. to detect LUTD. However, UDI is a non-physiological examination and may result in investigation-induced pathological findings. Indeed, in more than 70% of our healthy subjects, UDI revealed pathological findings, most commonly detrusor sphincter dyssynergia. Thus, UDI seems not to be a sensible tool to define normal lower urinary tract function and may result in exclusion of many potential candidates due to false positive pathological findings. Based on the current study, we do not recommend UDI to select healthy subjects as controls for comparative studies but rather rely on validated questionnaires, bladder diaries, free uroflowmetry, PVR, medical history and physical examination.

## Supporting Information

S1 File STROBE Statement Checklist. (DOC)

S2 File TREND Statement Checklist. (PDF)

S3 File Study protocol. (PDF)

S4 File Translation of the study protocol relevant parts. (DOCX)
