# Supplementary material for: Urodynamic Investigation: A Valid Tool to Define Normal Lower Urinary Tract Function?
Source: PLoS One. 2016 Oct 13;11(10):e0163847. doi: 10.1371/journal.pone.0163847 (PMC5063299; doi:10.1371/journal.pone.0163847)
Supplement: S3 File — (PDF) [file pone.0163847.s003.pdf]

Studienprotokoll

1 Titel der Studie

Untersuchung der supraspinalen Steuerung des unteren Harntraktes bei Patientinnen mit neurogener und nicht-neurogener Harnblasenfunktionsstörung

Untersuchung der Steuerung der Funktion der Harnblase durch das Gehirn beim Menschen

Versionsdatum:

1.1 Name des Prüfers

PD Dr. Thomas M. Kessler
Neuro-Urologie
Paraplegikerzentrum & Forschung
Universität Zürich
Uniklinik Balgrist
Forchstrasse 340
8008 Zürich
Schweiz

Unterschrift:

1.2 Liste der Mitarbeiter

Dr. Ulrich Mehnert
Neuro-Urologie
Paraplegikerzentrum & Forschung
Universität Zürich
Uniklinik Balgrist
Forchstrasse 340
8008 Zürich
Schweiz

Dr. Lars Michels
UniversitätsSpital Zürich
Institut für Neuroradiologie
Dept. Medizinische Radiologie
Rämistrasse 100
8091 Zürich
Schweiz

Dr. Dorothee Birnböck
Neuro-Urologie
Paraplegikerzentrum & Forschung
Universität Zürich
Uniklinik Balgrist
Forchstrasse 340
8008 Zürich
Schweiz

Prof. Dr. Spyros Kollias
UniversitätsSpital Zürich
Institut für Neuroradiologie
Dept. Medizinische Radiologie
Rämistrasse 100
8091 Zürich
Schweiz

2 Zielsetzungen und Zweck

Ziel dieser Studie ist es, die supraspinalen[1] neuronalen Steuerungsvorgänge zur Kontrolle des unteren Harntraktes und deren Veränderung bei Patientinnen mit Dysfunktion des unteren Harntraktes zu erfassen und damit besser zu verstehen. Es sollen mit dieser Studie neue Einblicke in die supraspinale Steuerung des unteren Harntraktes gewonnen werden, um bisherige theoretische Modelle zu bestätigen oder zu widerlegen.

2.1 Hintergrund, Begründung und Ziel der Studie

Funktionsstörungen des unteren Harntraktes mit oder ohne neurogene Schädigung als deren Ursache haben eine hohe Prävalenz, erzeugen eine starke Einschränkung der Lebensqualität für die Betroffenen und sind eine enorme ökonomische Belastung für jedes Gesundheitssystem (Heidler et al., 2007; Klotz et al., 2007; Wennberg et al., 2009).
Obwohl es bereits verschiedene Modelle und Theorien zur Funktion des unteren Harntraktes und dessen neuronaler Steuerung sowohl unter normalen als auch krankhaften Bedingungen gibt, sind die zugrunde liegenden genauen pathophysiologischen Vorgänge und Mechanismen beim Menschen weitgehend unbekannt (Fowler et al., 2008; Fowler and Griffiths, 2010).
Damit der untere Harntrakt, bestehend aus Harnblase, Blasenhals, Harnröhre und Harnröhrenschliessmuskel, korrekt funktionieren kann, sind diese Strukturen auf eine intakte neuronale Innervation unter der Kontrolle eines komplexen supraspinalen Netzwerkes angewiesen (Fowler et al., 2008; Fowler and Griffiths, 2010). Die Abhängigkeit des unteren Harntraktes von diesen komplexen supraspinalen Schaltkreisen macht ihn einzigartig im Vergleich zu anderen viszeralen Organen (z.B. Magen, Dünndarm, Herz-Kreislauf), aber auch anfälliger für neurologische Störungen.

---

[1] Supraspinal bedeutet oberhalb des Rückenmarks (Medulla spinalis) gelegen und bezeichnet in der Terminologie die topographische Lage des Gehirns.

Die funktionelle Bildgebung neuronaler Vorgänge und Strukturen mittels funktioneller Magnetresonanztomographie ist daher ein sehr geeignetes und darüber hinaus minimalinvasiv Verfahren, um supraspinale Vorgänge bei normaler Funktion und bei Dysfunktion des unteren Harntraktes zu untersuchen.
Im letzten Jahrzehnt haben erste Studien mit funktioneller Neurobildgebung einen fundamentalen Beitrag zum Grundverständnis der supraspinalen neuronalen Kontrolle/Steuerung des unteren Harntraktes zunächst bei gesunden Probanden, später auch vereinzelt bei Patienten, geleistet (Fowler and Griffiths, 2010). Dennoch bleiben viele Fragen offen, insbesondere bei Funktionsstörungen des unteren Harntraktes.
Es ist wenig über die Veränderungen der supraspinalen neuronalen Aktivität und Konnektivität in Bezug auf die Kontrolle des unteren Harntraktes bei Patienten mit nicht-neurogener Harnblasenüberaktivität im Vergleich zu Gesunden bekannt. Es ist gänzlich unbekannt, ob solche Veränderungen der supraspinalen Harntraktkontrolle durch Behandlungen (z.B. Botulinumtoxin Injektionen in den Harnblasenmuskel) moduliert bzw „normalisiert“ werden und ob diese „Normalisierung“ mit dem klinisch-anamnestischen Erfolg der Behandlung korreliert.
Es existieren keine Kenntnisse über die supraspinale neuronale Aktivität und Konnektivität in Bezug auf die Kontrolle des unteren Harntraktes bei Patienten mit Multipler Sklerose, die bekanntermassen häufig unter Funktionsstörungen des unteren Harntraktes leiden (Araki et al., 2003; de Seze et al., 2007).
Es gibt Hinweise, aber wenige Informationen über die Rolle der afferenten C-Fasern des unteren Harntraktes in den pathophysiologischen Vorgängen/Veränderungen bei Patienten mit Harnblasenüberaktivität (Jiang et al., 2002; Silva et al., 2002).
Die Untersuchung dieser Zusammenhänge und Erkenntnislücken, soll das Wissen über die neuronale Steuerung des unteren Harntraktes beim Menschen erweitern, um neue Therapien zu entwickeln und zu evaluieren und bekannte Therapien zu verbessern.

Ziel der Studie ist es daher, mittels funktioneller Magnetresonanztomographie:

1) Die Untersuchung der supraspinalen neuronalen Kontrolle des unteren Harntraktes bei Patientinnen mit nicht-neurogener Harnblasenüberaktivität, der Vergleich zu Gesunden ohne Harnblasenfunktionsstörungen und die Analyse möglicher charakteristischer/spezifischer Unterschiede zwischen Gesunden und Patientinnen mit nicht-neurogener Harnblasenüberaktivität in Bezug auf die supraspinale neuronale Aktivität und Konnektivität bei der Wahrnehmung und Kontrolle des unteren Harntraktes.
2) Die Untersuchung eines möglichen Einflusses einer Behandlung der Harnblasenüberaktivität (z.B. Botulinumtoxin Injektionen in den Harnblasenmuskel) auf die veränderte supraspinale Harnblasenkontrolle bei Patientinnen mit nicht-neurogener Harnblasenüberaktivität.
3) Die Untersuchung der supraspinalen neuronalen Kontrolle bei Patientinnen mit Harnblasenfunktionsstörungen im Rahmen einer Multiplen Sklerose (MS) und die Analyse möglicher charakteristischer/spezifischer Unterschiede zwischen Gesunden ohne Harnblasenfunktionsstörungen, Patientinnen mit nicht-neurogener Harnblasenüberaktivität und Patientinnen mit Harnblasen-funktionsstörungen durch MS in Bezug auf die supraspinale Aktivität und Konnektivität bei der Wahrnehmung und Kontrolle des unteren Harntraktes.
4) Die Untersuchung eines möglichen Einflusses abnormaler C-Faser Sensibilität auf den Pathomechanismus der Harnblasenüberaktivität beim Menschen.

2.2 Fragestellung, Studienpopulation

1) Fragestellung: Welche supraspinale neuronale Aktivität und Konnektivität lässt sich bei Patientinnen mit nicht-neurogener Harnblasenüberaktivität während repetitiver Harnblasenfüllung und –entleerung mittels funktioneller Magnetresonanztomographie des Gehirns feststellen? Welche Unterschiede bestehen im Vergleich zu gesunden Probanden?

   Studienpopulation: 10 Frauen im Alter zwischen 18 und 55 Jahren mit nicht-neurogener Harnblasenüberaktivität (d.h. keine neurologische Grunderkrankung oder neurologischer Schaden als Erklärung für die Harnblasenüberaktivität bekannt oder feststellbar) sowie 10 gesunde Frauen im Alter zwischen 18 und 55 Jahre ohne Beschwerden oder Funktionsstörungen des unteren Harntraktes (= gesunde Kontrollen).

2) Fragestellung: Wird durch eine Therapie der Harnblasenüberaktivität (z.B. Botulinumtoxin-Injektionen in den Harnblasenmuskel), die die Patientinnen von Ihrem behandelnden Arzt studienunabhängig erhalten, die supraspinale neuronale Aktivität und Konnektivität in Bezug auf die Wahrnehmung und Kontrolle des unteren Harntraktes verändert? Normalisieren sich mögliche pathologische neuronale Aktivitäten und Verbindungen und korreliert dies mit der klinischen Besserung der Symptome?

   Studienpopulation: 10 Frauen im Alter zwischen 18 und 55 Jahren mit nicht-neurogener Harnblasenüberaktivität (d.h. keine neurologische Grunderkrankung oder neurologischer Schaden als Erklärung für die Harnblasenüberaktivität bekannt oder feststellbar), die von ihrem behandelnden Arzt (meist Gynäkologe oder Urologe) für eine Behandlung mit Botulinumtoxin Injektionen in den Harnblasenmuskel vorgesehen sind. Diese Studienpopulation kann dieselbe wie bei Fragestellung 1 sein oder sich zumindest überschneiden.

   <u>Wichtig</u>: Das Patientenkollektiv für diese Studie besteht aus Patientinnen, bei denen aus medizinischen Gründen die Indikation für die Botulinumtoxin-Injektion gestellt wurde. Die Kostengutsprache mit der Krankenkasse bzw. den Patientinnen (s. 9.3) wurde bereits abgeklärt und die in Frage kommenden Patientinnen haben bereits ein Aufgebot für die Injektion erhalten. Dann erst werden sie für die Studienteilnahme angefragt. Das bedeutet, dass die Patientinnen die Therapie mit Botulinumtoxin völlig unabhängig von dieser Studie erhalten und auch unabhängig von ihrer Teilnahme oder Nicht-Teilnahme an dieser Studie. Der Studienteil besteht aus den fMRI Scans.

3) Fragestellung: Welche Unterschiede in der supraspinalen neuronalen Aktivität und Konnektivität in Bezug auf die Wahrnehmung und Kontrolle des unteren Harntraktes bestehen zwischen MS-Patientinnen mit und ohne Harnblasenüberaktivität?

   Studienpopulation: 10 Frauen im Alter zwischen 18 und 55 Jahren, bei denen MS diagnostiziert wurde. Die eine Hälfte der Studienpopulation hat auf Grund der Erkrankung eine Harnblasenüberaktivität entwickelt, die andere Hälfte hat trotz MS keine Harnblasenüberaktivität entwickelt.

4) Fragestellung: Lässt sich durch C-Faser-Stimulation ein pathologisches supraspinales Aktivitäts- und/oder Konnektivitätsmuster bei Patientinnen mit neurogener und/oder nicht-neurogener Harnblasenüberaktivität provozieren bzw. repoduzieren?

   Studienpopulation: Diese Fragestellung wird bei allen zuvor genannten Studienpopulationen der Fragestellungen 1-3 untersucht.

2.3 Hypothese

Defizite in der strukturellen/anatomischen und funktionellen neuronalen Konnektivität zwischen supraspinalen Bereichen, die für die Kontrolle des unteren Harntraktes verantwortlich sind, verursachen pathologische Veränderungen in der Wahrnehmung von, Verarbeitung von und Reaktion auf afferente Informationen vom unteren Harntrakt, die sich klinisch als Pollakisurie, Dysurie und/oder imperativer, überfallsartiger Harndrang äussern können (Tadic et al., 2010a; Tadic et al., 2010b; Tadic et al., 2008; Zempleni et al., 2010).
Mit der Verwendung von funktioneller Magnetresonanztomographie in Verbindung mit Diffusions-Tensor-Bildgebung[2] und funktioneller Konnektivitätsanalyse können die Defizite oder Veränderungen aufgedeckt werden, die zu einer Beeinträchtigung der neuronalen Konnektivität innerhalb des supraspinalen Netzwerks führen, das den unteren Harntrakt steuert. Obwohl diese Defizite in der neuronalen Konnektivität bei Patientinnen mit nicht-neurogener Harnblasenüberaktivität im Ruhezustand nicht unbedingt sichtbar gemacht werden können, so werden sie doch evident während der Untersuchungen mit Stimulation des unteren Harntraktes (z.B. durch Füllung der Harnblase mit körperwarmer und kalter Kochsalzlösung – siehe Versuchsdurchführung).
Bei den Patientinnen mit überaktiver Harnblase, die eine studienunabhängige Behandlung mit Botulinumtoxin-Injektion in den Harnblasenmuskel erhalten haben, wird sich eine deutliche Reduzierung von pathologischen neuronalen Aktivitäten und Verbindungen zeigen und eine Annäherung an das Aktivitäts- und Konnektivitätsmuster der gesunden Probanden festzustellen sein.
Patientinnen mit MS können durch die neurologische Grunderkrankung signifikante Veränderungen in der neuronalen Konnektivität bereits im Ruhezustand aufweisen. Während der Harnblasenfüllung können MS-Patientinnen ohne Harnblasenüberaktivität eine ähnliche supraspinale Aktivität und Konnektivität

---

[2] Die Diffusions-Tensor-Bildgebung (abgekürzt DTI von engl. *diffusion tensor imaging* oder DT-MRI von *diffusion tensor magnetic resonance imaging*) ist eine häufig eingesetzte Variante der DW-MRI, Die diffusionsgewichtete Magnetresonanztomografie (abgekürzt DW-MRI von engl. *diffusion-weighted magnetic resonance imaging*) ist ein bildgebendes Verfahren, das mit Hilfe der Magnetresonanztomografie (MRT) die Diffusionsbewegung von Wassermolekülen in Körpergewebe misst und räumlich aufgelöst darstellt. Sie wird in erster Linie zur Untersuchung des Gehirns eingesetzt, da das Diffusionsverhalten im Gewebe sich bei einigen Erkrankungen des zentralen Nervensystems charakteristisch verändert und die Richtungsabhängigkeit der Diffusion Rückschlüsse auf den Verlauf der großen Nervenfaserbündel erlaubt. Wie die klassische MRT ist die diffusionsgewichtete Bildgebung nichtinvasiv: Sie erfordert keine Kontrastmittel und verwendet keine potenziell schädliche ionisierende Strahlung.

zeigen, wie sie bei den gesunden Probandinnen zu sehen ist, alternativ lassen sich bereits ausgeprägte kompensatorische Aktivitäten und neuronale Verbindungen zu akzessorischen supraspinalen Arealen feststellen, die ein Aufrechterhalten der Funktion des unteren Harntraktes ermöglichen. MS-Patientinnen mit Harnblasenüberaktivität werden weder „normale“ noch kompensatorische supraspinale Aktivierung oder Konnektivität zeigen.
C-Faser Stimulation durch Infusion von kalter Kochsalzlösung in die Harnblase wird eine ähnliche oder verstärkte aberrante supraspinale Aktivität und Konnektivität hervorrufen wie sie bei Harnblasenfüllung in Patientinnen mit Harnblasenüberaktivität zu beobachten ist.

3. Studiendesign

3.1. Hauptzielparameter und sekundäre Zielparameter

Hauptzielparameter: Veränderung des blood-oxygen-level-dependent (BOLD) [3] Signals (in %) in Bezug auf supraspinaler Lokalisation (z.B. Pons, Insula, anteriorer Gyrus cinguli, Thalamus, akzessorischer Motorkortex, präfrontaler Kortex) und Intensität während niedrigem Harnblasenvolumen, hohem Harnblasenvolumen mit Harndrang und Kältestimulation der Harnblase bei der letzten Visite.
Das BOLD Signal wird bei allen Visiten (ausser Visite 1) mittels funktioneller Magnetresonanztomographie in einem 3T Philipps Achieva MR-Tomographen gemessen.

Sekundäre Zielparameter: Veränderung des blood-oxygen-level-dependent (BOLD) Signals in % in Bezug auf supraspinaler Lokalisation (z.B. Pons, Insula, anteriorer Gyrus cinguli, Thalamus, akzessorischer Motorkortex, präfrontaler Kortex) und Intensität während niedrigem Harnblasenvolumen, hohem Harnblasenvolumen mit Harndrang und Kältestimulation der Harnblase bei Visite 2 und ggf. Visite 3.
Strukturelle/anatomische (Faserdichte und Faseranisotropie) und funktionelle (zeitliche Korrelation der neuronalen Aktivität) Konnektivität zwischen supraspinalen Zentren, die in die Steuerung des unteren Harntraktes bekanntermassen involviert sind. Dies betrifft insbesondere Verbindungen zwischen präfrontalem Kortex, Thalamus, Insula und anteriorem Gyrus cinguli.
Die supraspinale Faserdichte und Faseranisotropie wird bei allen Visiten (ausser Visite 1) mittels Diffusions-Tensor-Bildgebung in einem 3T Philipps Achieva MR-Tomographen gemessen.
Die zeitliche Korrelation der neuronalen Aktivität wird aus den fMRT-Daten mittels spezieller Software (z.B. SPM8, Brain connetivity tool box) berechnet.

[3] Als BOLD-Kontrast (von englisch *blood oxygenation level dependent*, also „abhängig vom Blutsauerstoffgehalt“) bezeichnet man in der Magnetresonanztomographie (MRT) die Abhängigkeit des (Bild-)Signals vom Sauerstoffgehalt in den roten Blutkörperchen. Die Hauptanwendung des BOLD-Kontrasts ist die funktionelle MRT (fMRT) zur Darstellung der Hirnaktivität. Synonym wird das Akronym BOLD auch für *blood oxygen level dependent* oder (seltener) *blood oxygen(ation) level dependence/dependency* verwendet

3.2. Studiendesign und Studienablauf, Abbruchkriterien

Studiendesign: Monozentrische, nicht randomisierte, kontrollierte Grundlagenstudie an mindestens 40 lebenden Menschen, die über einen Zeitraum von 36 Monaten geplant ist. Verlängerung bei unzureichender Probandenrekrutierung möglich.

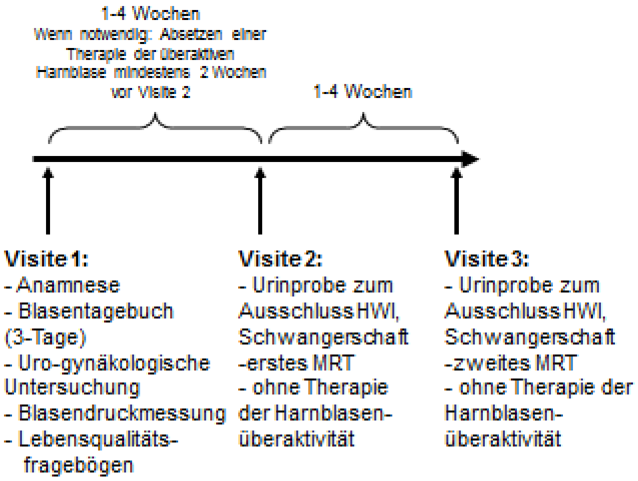

Abb. 1: Schematischer Studienablauf

Studienablauf:
Alle Studienteilnehmerinnen sollen an drei Visiten teilnehmen (Abb. 1). Während Visite 1 erfolgt eine ausführliche Anamnese und Listung aller ggf eingenommenen Medikamente. Die Teilnehmerinnen werden auf ihre Eignung für die Untersuchung im Magnetresonanztomographen überprüft (z.B. kein ferromagnetisches Metall im Körper, kein Herzschrittmacher, keine Raumangst, Ausfüllen der Unterlagen (z. B. fMRI Sicherheitsbogen, Händigkeitstest, s. Anlagen)). Zudem werden eine uro-gynäkologische Untersuchung und eine Füllzystometrie (= Blasendruckmessung) zur Sicherung der Diagnose gemäss der Ein- und Ausschlusskriterien durchgeführt.
Bei der urogynäkologischen Untersuchung wird die Patientin in Steinschnittlage vaginal eingestellt, d.h. mittels Spekulum, und digitaler Tastuntersuchung wird Trophik und Geschmeidigkeit der Vagina beurteilt, sowie auf Cysto- oder Rektozelen und Entzündungen im Urogenitalbereich untersucht. Ein Hustentest zum Ausschluss einer Belastungsinkontinenz gehört ebenfalls dazu.
Bei einer Füllzystometrie: wird die Blase mit einer definierten Füllgeschwindigkeit über ein Kathetersystem aufgefüllt und dabei kontinuierlich der intravesikale sowie der näherungsweise über eine rektale Drucksonde gemessene intraabdominale Druck gemessen. Durch Subtraktion des intravesikalen vom intraabdominalen Druck kann somit der Detrusordruck ermittelt werden. Es werden zumindest die Füllmenge und der Detrusordruck zum Zeitpunkt des ersten Harndrangs aufgezeichnet sowie eine Aussage über die maximale Blasenkapazität getroffen. Die Füllzystometrie kann durch den Nachweis isolierter Detrusorkontraktionen oder abnorm hoher Blasendruckwerte im Zusammenhang mit entsprechenden Symptomen der Patientin eine Detrusorüberaktivität belegen bzw. ausschließen. Aus demselben Grund werden die Teilnehmer gebeten, ein Blasentagebuch (s. Anlage) über 3 Tage zu führen und einen Lebensqualitätsfragebogen, der spezifisch für Patienten mit Harnblasenüberaktivität konzipiert und validiert ist, auszufüllen.

Das Blasentagebuch und den Lebensqualitätsfragebogen (The International Consultation on Incontinence Questionnaire (ICIQ), The OAB-q, s. Anlage) retournieren die Teilnehmerinnen per frankiertem Rückumschlag innert einer Woche. Erfüllen die Teilnehmerinnen sämtliche Einschlusskriterien und keine Ausschlusskriterien, werden sie für Visite 2 einbestellt.
Sollten Patientinnen mit einer Harnblasenüberaktivität eine antimuskarinerge Therapie erhalten, werden sie gebeten, mit dieser mindestens 2 Wochen vor Visite 2 zu pausieren.

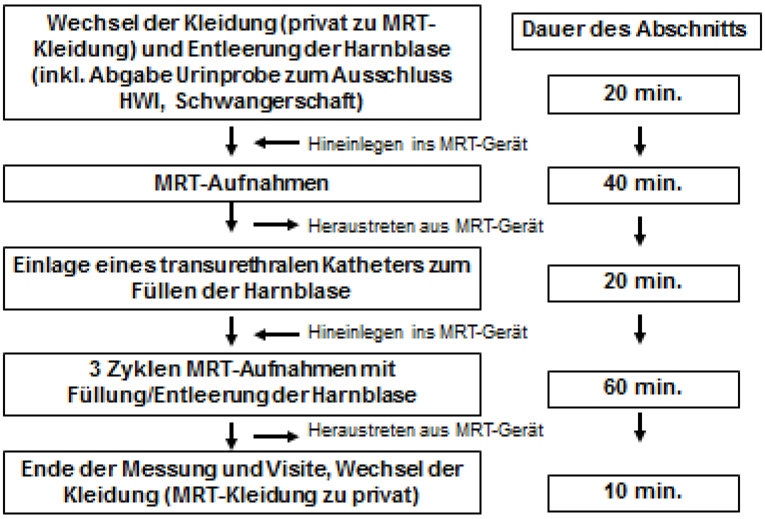

Abb. 2: Schematische Übersicht zum Ablauf der Untersuchung im MR-Zentrum bei Visite 2, 3 und ggf. 4.

Während Visite 2 erfolgt die erste fMRT-Untersuchung (Abb. 2 und 3). Alle fMRT-Untersuchungen finden im MR-Zentrum des UniversitätSpitals Zürich an einem 3T Philipps Achieva MR-Tomographen statt. Damit nicht versehentlich ferromagnetische Gegenstände (z.B. Schmuck, Geld, Schlüssel, Reissverschlüsse, BH-Bügel) oder magnetisch empfindliche Gegenstände (z.B. Kreditkarten) mit der privaten Kleidung in den MRT-Raum gelangen, werden alle Teilnehmerinnen gebeten, in einer separaten Umkleidekabine ihre private Kleidung, Schmuck und sonstige Gegenstände abzulegen und die bereitgestellten MRT-tauglichen Stoffhosen und –kasaks zu benutzen.
Sodann entleeren alle Teilnehmerinnen ihre Harnblase, um eine Urinprobe abzugeben und damit die Aufnahmen im Ruhezustand bei leerer Harnblase stattfinden können. Die Urinprobe ist notwendig, um mittels entsprechendem Teststreifen Combur Stix® einen Harnwegsinfekt oder eine Schwangerschaft (Clearblue®) bei den Teilnehmerinnen auszuschliessen.
Sobald die Teilnehmerinnen bequem im fMRT-Gerät liegen, den Gehörschutz erhalten haben und über die Alarmknopffunktion instruiert worden sind, wird mit den Messungen begonnen (Abb. 2). Zunächst werden die strukturellen/anatomischen MR-Aufnahmen gemacht, anschliessend die Diffusions-Tensor-Bildgebung und danach die fMRT-Aufnahmen im Ruhezustand. Die Reihenfolge der Aufnahmen kann variieren. Dann verlassen die Teilnehmerinnen zunächst das MRT-Gerät, damit ein transurethraler Katheter eingelegt werden kann. Der Katheter dient bei den nachfolgenden fMRT-Aufnahmen zur raschen und kontrollierten Harnblasenfüllung und Entleerung (Abb. 3).
Nach Kathetereinlage legen sich die Teilnehmerinnen wieder ins MRT-Gerät und es folgen 3 Messzyklen, die aus je 10 Wiederholungen verschiedener, aber in fester

Reihenfolge ablaufender Bedingungen (z.B. REST = Nichts passiert, Filling = Harnblase wird mit 100 ml gefüllt, DRAIN = Harnblase wird entleert) bestehen (Abb. 3).
Vor Messzyklus 1 wird die Harnblase mit 100 ml vorgefüllt. Vor Messzyklus 2 wird die Harnblase gefüllt, bis die Teilnehmerin einen starken Harndrang angibt (Abb. 3). Vor Messzyklus 3 wird die Harnblase komplett entleert (Abb. 3). Die Füllung und Entleerung der Harnblase bei Messzyklus 1 und 2 mit steriler körperwarmer Kochsalzlösung wird manuell mit einer 100 ml Blasenspritze durchgeführt. Bei Messzyklus 3 wird die kalte Kochsalzlösung passiv in die Harnblase infundiert und ebenfalls passiv abgelassen.

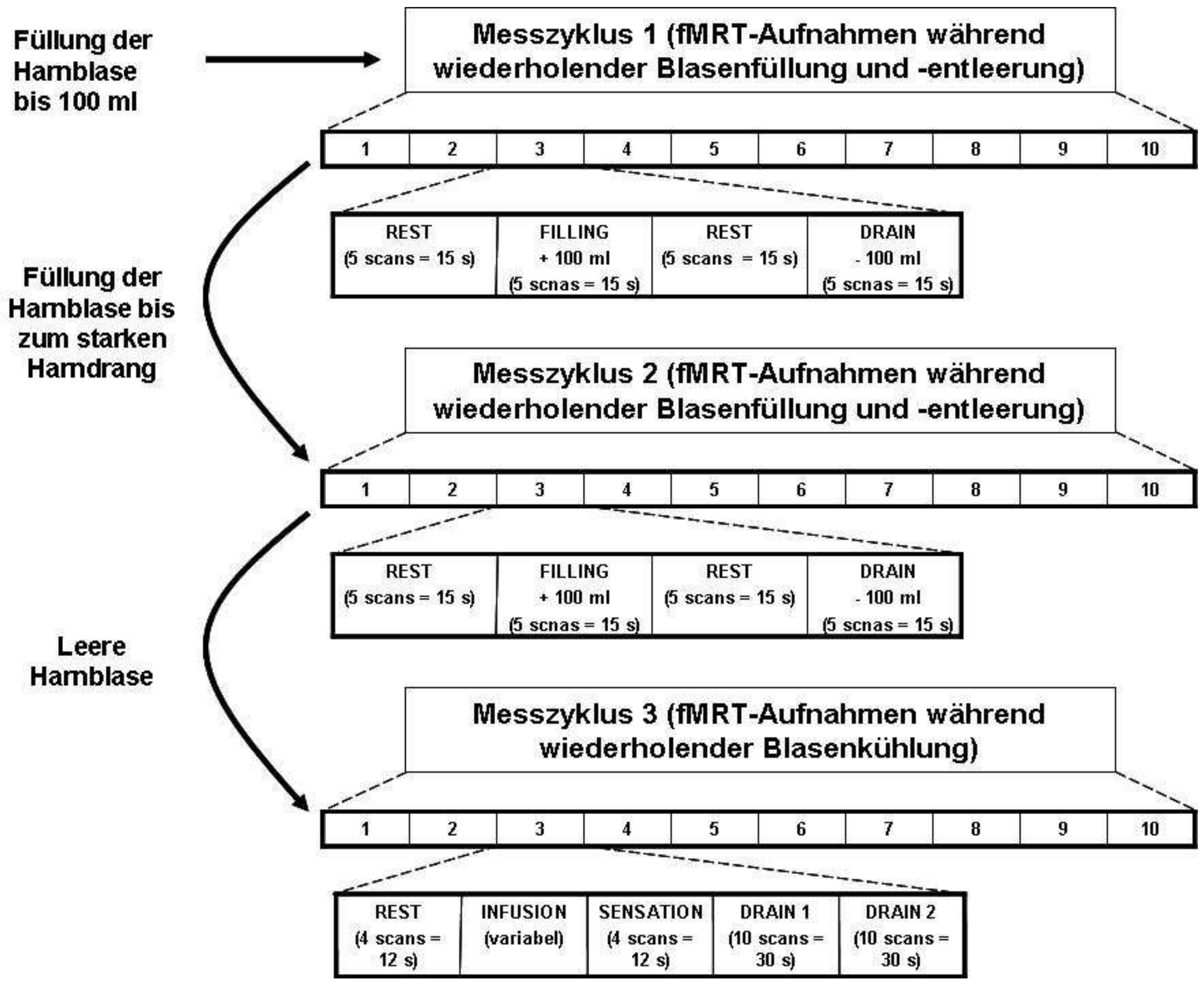

Abb. 3: schematische Detaildarstellung des Ablaufs der einzelnen Messzyklen im MRT-Gerät bei Visite 2, 3 und ggf. 4.

Nach Beendigung von Messzyklus 3 wird der transurethrale Katheter entfernt und die Teilnehmerinnen können das MRT-Gerät verlassen und die Privatkleidung anlegen. Zum Schluss geben die Teilnehmerinnen noch mittels einer visuellen Analogskala (0 bis 10) an, wie angenehm bzw. unangenehm die Untersuchung für sie war. Die Visite 2 ist damit abgeschlossen.
Visite 3 folgt im Abstand von 1 bis 4 Wochen und verläuft in gleicher Weise wie Visite 2.
Wird eine Patientin mit nicht-neurogener Harnblasenüberaktivität im Verlauf nach Visite 3 von ihrem behandelnden Urologen/Gynäkologen auf Grund ihrer Beschwerden mit Botulinumtoxin-Injektionen in den Harnblasenmuskel behandelt, wird sie zu einer zusätzlichen 4. Visite eingeladen (Abb. 4). Visite 4 erfolgt 5 bis 7 Wochen nach der Behandlung mit Botulinumtoxin und verläuft in gleicher Weise wie

Visite 2 und 3. Zusätzlich wird bei Visite 4 noch eine Füllzystometrie durchgeführt und die Teilnehmerinnen füllen wie im Rahmen von Visite 1 ein Blasentagebuch (währen 3 Tagen) und einen Lebensqualitätsfragebogen aus (Abb. 4).

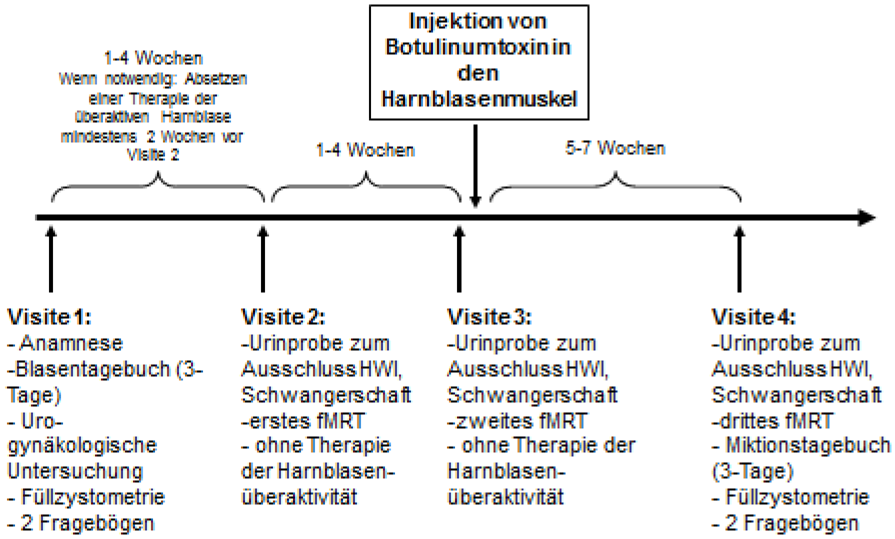

Abb. 4: Schematische Darstellung des Studienablaufs mit einer zusätzlichen 4. Visite bei Patientinnen mit nicht-neurogener Harnblasenüberaktivität, die von ihrem behandelnden Urologen/Gynäkologen Botulinumtoxin Injektionen in den Harnblasenmuskel zur Therapie der Harnblasenüberaktivität erhalten.

Alle beschriebenen Untersuchungen, die während aller Visiten erfolgen, werden von medizinisch geschultem Personal, einem der aufgeführten Mitarbeiter oder dem Studienleiter durchgeführt.
Der zeitliche Aufwand für die Studienteilnehmerinnen beträgt etwa 2 1/2 Stunden pro Visite.

<u>Abbruchkriterien:</u>

- Schmerzen durch die Untersuchungen/Messungen
- Raumangst im MRT-Gerät
- neu eingetretene Schwangerschaft
- Pausierung einer allfälligen antimuskarinergen Therapie der Harnblasenüberaktivität wird nicht toleriert
- jegliche Umstände, bei denen es durch die Studienteilnahme zu einer Gefährdung der Gesundheit der Studienteilnehmer kommt
- Studienteilnehmer hält sich nicht an die Anweisungen des Studienarztes

3.3 Massnahmen zur Bias-Minimierung

Zur Minimierung von Biases und zur Überprüfung der Reliabilität der Messdaten durchlaufen alle Studienteilnehmerinnen zwei fMRT-Untersuchungen (Visite 2 und 3). Die Studienteilnehmerinnen werden nach genau definierten Ein- und Ausschlusskriterien ausgewählt. Die gesunde Kontrollgruppe ist mit der Gruppe der Patientinnen mit nicht-neurogener Harnblasenüberaktivität altersgematcht.
Es wird eine Gruppen-, als auch Einzelpersonen-Analyse der fMRT-Daten durchgeführt, um die Gewichtung der BOLD Signal Intensität besser einschätzen zu

können, d.h. um zu erkennen, ob das Gruppenresultat nur von starken Aktivierungen einzelner Teilnehmer resultiert oder durch gleichmässige Aktivierung bei vielen Teilnehmern. In jedem Fall wird eine statistische Korrektur mittels false discovery rate (FDR) durchgeführt.
Um einen Bias durch Kopfbewegungen auszuschliessen bzw zu minimieren, werden die fMRT-Daten aller Teilnehmer einer individuellen Bewegungskorrektur unterzogen. Messungen mit sehr starken Kopfbewegungen (> 1 cm) werden von der Analyse ausgeschlossen.

4 Auswahl von Versuchspersonen

4.1 Rekrutierung

Die Rekrutierung der gesunden Probandinnen erfolgt durch Aushang und/oder Annonce (s. Anlage).
Die Rekrutierung der Patientinnen mit nicht-neurogener Harnblasenüberaktivität erfolgt im Rahmen ihrer Vorstellung in der urologischen oder gynäkologischen Sprechstunde in der Urologischen oder Gynäkologischen Klinik des UniversitätsSpitals Zürich.
Die Rekrutierung der MS-Patientinnen erfolgt im Rahmen ihrer Vorstellung in der neurologischen Sprechstunde der Neurologischen Klinik des UniversitätsSpitals Zürich oder in der neuro-urologischen Sprechstunde des Paraplegikerzentrums der Universität Zürich an der Uniklinik Balgrist.

4.2 Einschlusskriterien

Gemeinsame Einschlusskriterien für gesunde Probandinnen, Patientinnen mit nicht-neurogener Harnblasenüberaktivität, MS-Patientinnen ohne und MS-Patientinnen mit Harnblasenüberaktivität:

- Weiblich
- 18-55 Jahre
- MR-Tauglichkeit
- negativer Schwangerschaftstest
- schriftliches Einverständnis zur Studienteilnahme

4.2.1 Einschlusskriterien für gesunde Probandinnen

- 18-55 Jahre (altersmatching zu Patientinnen mit nicht-neurogener Harnblasenüberaktivität)
- Gesund
- Unbeeinträchtigte Funktion des unteren Harntraktes

4.2.2 Einschlusskriterien für Patientinnen mit nicht-neurogener Harnblasenüberaktivität

- Nicht-neurogene Harnblasenüberaktivität seit > 6 Monaten (mit ≥ 3 Episoden imperativem, überfallsartigem Harndrang und Pollakisurie > 8/24h im 3-Tages-Blasentagebuch)

4.2.3 Einschlusskriterien für MS-Patienteninnen ohne Harnblasenüberaktivität

- Primäre progressive MS (nach McDonald Kriterien)[4] seit ≥ 1 Jahr
- EDSS[5] ≤ 6

4.2.4 Einschlusskriterien für MS-Patientinnen mit Harnblasenüberaktivität

- neurogene Harnblasenüberaktivität seit > 6 Monaten (mit ≥ 3 Episoden imperativem, überfallsartigem Harndrang und Pollakisurie > 8/24h im 3-Tages-Blasentagebuch)
- Primäre progressive MS (nach McDonald Kriterien)[6] seit ≥ 1 Jahr
- EDSS[7] ≤ 6

4.3. Ausschlusskriterien

Gemeinsame Ausschlusskriterien für gesunde Probandinnen, Patientinnen mit nicht-neurogener Harnblasenüberaktivität, MS-Patientinnen ohne und MS-Patientinnen mit Harnblasenüberaktivität:

- Männlich
- Schwangerschaft
- Operationen oder Verletzungen an Schädel oder Gehirn
- Ferromagnetische Implantate oder Herzschrittmacher
- Chronische/rezidivierende Harnwegsinfektionen
- Teilnahme an anderen Studien
- Klaustrophobie

4.3.1 Ausschlusskriterien für gesunde Probandinnen

- Neurologische oder psychologische Erkrankungen
- Operationen oder Erkrankungen des Urogenitaltraktes
- Fehlbildungen des Urogenitaltraktes

---

[4] McDonald-Kriterien: Kriterien zur Diagnose der MS, die neben klinischen die Bedeutung bildgebender Befunde (MRT) betont. Auch paraklinische Befunde (Liquordiagnostik und evozierte Potentiale) wurden berücksichtigt; sie verloren jedoch gegenüber bildgebenden Befunden an Bedeutung.

[5] Die "EDSS" ist eine Leistungsskala nach Kurzke J.F. (expanded disability status scale) und gibt Auskunft über den Grad der Behinderung eines MS-Patienten. Sie reicht von 0 (keine neurologischen Defizite) bis 10 (Tod infolge MS). Die Angaben der Grade (von 0-10) in der EDSS beziehen sich auf die Untersuchung der funktionellen Systeme (FS), durch den behandelnden Arzt.

[6] McDonald-Kriterien: Kriterien zur Diagnose der MS, die neben klinischen die Bedeutung bildgebender Befunde (MRT) betonen. Auch paraklinische Befunde (Liquordiagnostik und evozierte Potentiale) wurden berücksichtigt; sie verloren jedoch gegenüber bildgebenden Befunden an Bedeutung.

[7] Die "EDSS" ist eine Leistungsskala nach Kurzke J.F. (expanded disability status scale) und gibt Auskunft über den Grad der Behinderung eines MS-Patienten. Sie reicht von 0 (keine neurologischen Defizite) bis 10 (Tod infolge MS). Die Angaben der Grade (von 0-10) in der EDSS beziehen sich auf die Untersuchung der funktionellen Systeme (FS), durch den behandelnden Arzt.

- Metabolische Erkrankungen (z.B. Diabetes Mellitus)
- Symptome einer Harnblasenüberaktivität

4.3.2 Ausschlusskriterien für Patientinnen mit nicht-neurogener Harnblasenüberaktivität

- Neurologische oder psychologische Erkrankungen
- Operationen des Urogenitaltraktes
- Fehlbildungen des Urogenitaltraktes
- Metabolische Erkrankungen (z.B. Diabetes Mellitus)
- Belastungsinkontinenz
- Restharnwerte nach Miktion von > 150 ml
- Ein maximaler Harnstrahl bei Miktion von < 15 ml/s
- Urinableitung über Dauerkatheter oder Selbstkatheterismus

4.3.3 Ausschlusskriterien für MS-Patientinnen ohne Harnblasenüberaktivität

- Neurologische oder psychologische Erkrankungen ausser MS
- Operationen oder Erkrankungen des Urogenitaltraktes
- Fehlbildungen des Urogenitaltraktes
- Metabolische Erkrankungen (z.B. Diabetes Mellitus)
- Belastungsinkontinenz
- Restharnwerte nach Miktion von > 150 ml
- Ein maximaler Harnstrahl bei Miktion von < 15 ml/s
- Urinableitung über Dauerkatheter oder Selbstkatheterismus
- Symptome einer Harnblasenüberaktivität
- Detrusorüberaktivität

4.3.4 Ausschlusskriterien für MS-Patientinnen mit Harnblasenüberaktivität

- Neurologische oder psychologische Erkrankungen ausser MS
- Operationen des Urogenitaltraktes
- Fehlbildungen des Urogenitaltraktes
- Metabolische Erkrankungen (z.B. Diabetes Mellitus)
- Belastungsinkontinenz
- Restharnwerte nach Miktion von > 150 ml
- Ein maximaler Harnstrahl bei Miktion von < 15 ml/s
- Urinableitung über Dauerkatheter oder Selbstkatheterismus

5 Bewertung der Wirksamkeit

5.1 Wirksamkeitsparameter: Messmethoden und Zeitpunkte

Bei dieser Grundlagenstudie wird keine Bewertung von Wirksamkeit durchgeführt.

6 Bewertung der Sicherheit

6.1 Sicherheitsparameter

Art und Häufigkeit der vorgesehenen Überwachungsmassnahmen:

i. vor der Untersuchungsperiode:

- Anamnese bezüglich Vorerkrankungen und Medikamenteneinnahme
- gynäkologische Untersuchung

- Füllzystometrie und 3-Tages-Blasentagebuch zur korrekten Indikationsstellung

ii. während der Untersuchungsperiode

- Urinuntersuchung vor den Messungen
- Schwangerschaftstest vor den Messungen
- Notfallknopf während der fMRT-Aufnahmen

iii. nach der Untersuchungsperiode

- Telefonisches Interview durch die wissenschaftliche Mitarbeiterin Frau Dr. Birnböck über das Wohlbefinden und etwaige Nebenwirkungen.

6.2 Sicherstellung der Nachbeobachtung von Versuchspersonen nach unerwünschten Ereignissen

Die in dieser Studie vorgesehen Untersuchungen sind minimal invasiv und es sind keine schwerwiegenden Ereignisse zu erwarten. Die schwerwiegenste Nebenwirkung wäre eine Harnwegsinfektion im Rahmen der Füllzystometrie oder der Katheterisierung bei den fMRT-Untersuchungen.
Sollten trotz sorgfältiger Beachtung aller Vorsichtmassnahmen (Überprüfung auf MR-Tauglichkeit und auf Harnwegsinfektion vor jeder Untersuchung) dennoch unerwünschte Ereignisse eintreten, kann von dem Studienarzt, der stets bei allen Untersuchungen anwesend ist, eine sofortige Notfalltherapie eingeleitet werden. Das MR-Gerät verfügt über einen einfach zu bedienenden Notknopf, über den die Teilnehmer jederzeit eine Notfallsituation signalisieren können, um dann rasch aus dem MR-Gerät geholt zu werden. Das MR-Zentrum im UniversitätsSpital Zürich verfügt über einen Notfallwagen mit allen notwendigen Medikamenten und Instrumenten, um im Notfall die Kreislauf und Atmung zu stabilisieren. Durch die direkte Anbindung ans UniversitätsSpital kann sofort ein Notfallteam hinzugerufen werden und ggf. eine intensivmedizinische Versorgung eingeleitet werden.

7 Statistik

7.1 Definition des primären Endpunktes und der sekundären Endpunkte

<u>Hauptzielparameter:</u> Veränderung des blood-oxygen-level-dependent (BOLD) Signals (in %) in Bezug auf supraspinaler Lokalisation (z.B. Pons, Insula, anteriorer Gyrus cinguli, Thalamus, akzessorischer Motorkortex, präfrontaler Kortex) und Intensität bei der letzten Visite. Variablen sind Alter, das Harnblasenvolumen und die Harnblasensensitivität sowie der Aufmerksamkeitszustand.

<u>Sekundäre Zielparameter:</u> Strukturelle/anatomische (Faserdichte und Faseranisotropie) und funktionelle (zeitliche Korrelation der neuronalen Aktivität) Konnektivität zwischen supraspinalen Zentren, die in die Steuerung des unteren Harntraktes bekanntermassen involviert sind. Dies betrifft insbesondere Verbindungen zwischen präfrontalem Kortex, Thalamus, Insula und anteriorem Gyrus cinguli.
Variablen sind Alter, das Harnblasenvolumen und die Harnblasensensitivität sowie der Aufmerksamkeitszustand.
Die zeitliche Korrelation der neuronalen Aktivität wird aus den fMRT-Daten mittels spezieller Software (z.B. SPM8, Brain connetivity tool box) berechnet.

7.2 Geplante Anzahl Versuchspersonen mit nachvollziehbarer Begründung

Bei einer derartigen Grundlagenstudie ist eine Poweranalyse nicht möglich. Die Ergebnisse werden ja sowohl als Einzelpersonenanalyse als auch als Gruppenanalyse dargestellt. Jede Versuchsperson ist damit ihre eigene Kontrolle. Aus eigener Erfahrung und der bisherigen Literatur (Fowler and Griffiths, 2010; Griffiths and Tadic, 2008; Griffiths et al., 2007; Mehnert et al., 2008; Mehnert et al., 2010; Tadic et al., 2010a; Tadic et al., 2010b; Tadic et al., 2008; Zempleni et al., 2010) auf diesem Gebiet und im Bereich der funktionellen Neurobildgebung sind mindestens 10 Personen pro Gruppe auszuwerten (gesamt mindestens 40), um repräsentative und glaubhafte Gruppenresultate zu präsentieren.

7.3 Beschreibung der vorgesehenen statistischen Methoden und der geplanten Zwischenauswertungen

Nach dem Transfer und der Sicherung der fMRI-Rohdaten vom MR-Rechner, werden diese an einem separaten IT-Arbeitsplatz entweder mit BrainVoyager QX (Brain Innovation B.V., Maastricht, The Netherlands) oder Statistical Parametric Mapping Version 8 (SPM8) ausgewertet.
Die funktionellen Daten werden mittels Bewegungskorrektur, räumlicher Glättung, und linearer Trendentfernung zunächst vorverarbeitet.
Mit BrainVoyager werden die anatomischen 3D Bilder zur iso-voxel Grösse von 1x1x1 mm3 interpoliert und mit den funktionellen Daten co-registriert. Anschliessend werden die co-registrierten Datensätze in das Talairach Koordinatenraster transformiert (Talairach and Tournoux, 1988).
Mit SPM8 werden alle Daten der funktionellen Aufnahmen normalisiert und der Koordinaten-Schablone des Montreal Neurological Institute (MNI) angepasst.
Eine auf die komplexe Auswertung von fMRT-Daten angepasste statistische Analyse ist bereits sowohl in BrainVoyager QX als auch SPM8 integriert. Die dabei am meisten verwendeten statistischen Verfahren und Tests sind: ANOVA, ANCOVA, t-Test.

7.4 Geplantes Signifikanzniveau

Ein Signifikanzniveau von < 0.05 nach FDR-Korrektur oder < 0.01 ohne Korrektur werden verwendet. Dies sind akzeptierte Signifikanzniveaus im Bereich der Auswertung von Neurobildgebungsdaten.

7.5 Umgang mit fehlenden Daten sowie mit Daten bei vorzeitigem Studienabbruch von Teilnehmern

Bei unerwartetem Datenverlust (z. B. Absturz des MR-Servers) oder bei vorzeitigem Studienabbruch werden die bis zum Abbruch erhobenen Daten verwertet und nach Möglichkeit in die endgültige Analyse eingeschlossen. Bei zu unvollständigen Daten wird eine neue Patientin/Probandin rekrutiert.

7.6 Definition der Auswertungsgruppen

Es werden alle stationären und ambulanten Patientinnen angefragt, an der Studie teilzunehmen, falls sie die entsprechenden Einschlusskriterien erfüllen und alle Personen, die sich auf den Aushang bzw. die Annonce melden. Die Daten aller in die Studie eingeschlossenen Personen werden ausgewertet.
Siehe Punkt 4.2 und 4.3.

8 Studienspezifische Vorsichtsmassnahmen und Pflichten

Die Füllzystometrie ist eine etablierte und standardisierte Untersuchung zur Diagnose der Harnblasen- und Schliessmuskelfunktion. Das Katheterisieren wird unter sterilen Bedingungen durchgeführt. Der Katheter kann unangenehm für die Patientin während der Untersuchung sein. Eine Harnweginfektion ist die am meisten beschriebene Komplikation nach einer Füllzystometrie (7-10%). Andere Komplikationen wie eine Verletzung der Harnröhre oder Blase durch den weichen Katheter sind extrem selten.
Wenn die Sicherheitsvorschriften und Vorsichtsmassnahmen beachtet werden, ist das Auftreten von Nebenwirkungen bei einer fMRT-Messung höchst unwahrscheinlich. Implantiertes ferromagnetisches Material und/oder ein Herzschrittmacher sind Ausschlusskriterien. Raumangst kann gelegentlich eines der Probleme während der Messungen im MR-Tomographen sein. Alle Teilnehmerinnen werden vor der Untersuchung ausführlich befragt und füllen den fMRT Sicherheitsbogen (s. Anlage) aus. Sollte es während der fMRT-Messung zu plötzlicher Raumangst kommen, wird die Untersuchung abgebrochen und die Teilnehmerin aus dem MRT-Gerät entfernt. Da das MRT-Gerät während der Messungen Lärm verursacht, erhalten die Patientinnen Ohrstöpsel.
Das Absetzen einer möglicherweise bereits begonnenen Therapie der Harnblasenüberaktivität mit antimuskarinergen Medikamenten 2 Wochen vor der ersten fMRT-Messung, falls nicht ohnehin bereits wegen Wirkungslosigkeit erfolgt, dient zum Ausschluss einer Verfälschung unserer Studienresultate durch diese Therapie. Das Absetzen der Therapie bedeutet für die Teilnehmer keinerlei Gefährdung. Im schlimmsten Fall kann es lästig sein. Wird in seltenen Fällen ein Absetzen der Therapie nicht toleriert, kann der Teilnehmer die Studie abbrechen.
Patienten mit einer bekannten Neigung zu Harnwegsinfekten werden vor Untersuchungsbeginn antibiotisch abgedeckt bzw. Antibiogrammgerecht behandelt.
Die transurethrale Katheterisierung (sowohl zur Füllzystometrie als auch bei den fMRT-Messungen) wird nach sterilen Kautelen von medizinisch geschultem Personal vorgenommen. Vor der Untersuchung wird ein Harnwegsinfekt mit einem Urin-Schnelltest (Combur-Stix®) ausgeschlossen. Sollte zu diesem Zeitpunkt ein Harnwegsinfekt vorliegen oder kann dieser nicht sicher ausgeschlossen werden, erfolgt bei Vorliegen entsprechender Symptome die Verordnung eines Antibiotikums und die Untersuchung/der Eingriff wird verschoben, bis der Harnwegsinfekt ausgeheilt ist.
Die Patienten können sich bei Beschwerden und Fragen sowohl telefonisch, als auch durch direktes Aufsuchen der Ambulanz der Neuro-Urologie am Paraplegikerzentrum der Uniklinik Balgrist jederzeit an uns wenden.

8.2 Abschlussuntersuchung

Eine Abschlussuntersuchung ist bei den wenig invasiven und gutverträglichen Untersuchungen nicht vorgesehen. Nach Abschluss aller Untersuchungen wird ein

Telefoninterview zur Erfassung des Wohlbefindens und möglicher Nebenwirkungen durchgeführt.

9 Pflichten des Prüfers

9.1 Bestätigung

Es wird bestätigt, dass die Studie gemäss Protokoll, GCP und den geltenden gesetzlichen Bestimmungen durchgeführt wird.
Alle Studienteilnehmer werden sowohl schriftlich als auch in einem Aufklärungsgespräch über die Studie informiert.

Die Aufklärung umfasst Informationen:	Ja	Nein
über das Ziel der Studie	**x**	
über die Durchführung der Studie	**x**	
über den zu erwartenden Nutzen für die Versuchsperson und für die Wissenschaft	**x**	
über alle für die Versuchsperson relevanten Erkenntnisse, die im Laufe des Versuchs gewonnen werden	**x**	
über mögliche unerwünschte Nebenwirkungen und Komplikationen	**x**	
über Risiken	**x**	
über den Versicherungsschutz	**x**	
über das Recht, jederzeit Fragen zu stellen und Beschwerden zu äussern	**x**	
mit Hinweis über den vertraulichen Umgang mit den persönlichen Daten	**x**	
mit Hinweis auf eine Bedenkfrist	**x**	
mit Hinweis auf das Verweigerungsrecht	**x**	
mit Hinweis, dass die Teilnahme an der Studie jederzeit widerrufen werden kann	**x**	

❑

Die von der Teilnehmerin und Studienarzt unterschriebene und datierte schriftliche Einwilligungserklärung ist Voraussetzung vor Teilnahme an der Studie.

9.2 Berichterstattung

Berichte über unerwünschte und schwerwiegende unerwünschte Ereignisse, Protokolländerungen und Abschlussberichte werden an die zuständigen Stellen der Ethikkommission gesandt werden. Ausserdem werden sie in den case report forms und den Krankenakten festgehalten.

9.3 Stellungnahme zur Deckung von Schäden

Es entstehen keine zusätzlichen Kosten für die Patientinnen. Die Kosten zur Durchführung studienspezifischen Untersuchungen und Messungen werden durch Forschungsgelder finanziert.
Die Patientinnen sind für die studienunabhängige klinische Diagnostik und Therapie über die Klinikhaftpflicht der Uniklinik Balgrist versichert. Für allfällige studienspezifische Schäden sind die Studienteilnehmerinnen über eine

Probandenversicherung bei der AXA Winterthur (Versicherungspolice Nr. 14.050.565) versichert.

10 Ethische Überlegungen

10.1 Bewertung des Risiko-Nutzen Verhältnisses

10.1.1 Nutzen

	Nein	Möglich	Sehr wahrscheinlich
Besteht im Rahmen der Studie ein diagnostischer Wert für die Versuchspersonen?		**X** Alle Patienten	
Besteht im Rahmen der Studie ein therapeutischer Wert für die Versuchspersonen?	**X**		

10.1.2 Risiko

	Ja	Nein
Hohes gesundheitliches Risiko		**x**
Mäßiges gesundheitliches Risiko		**x**
Geringes gesundheitliches Risiko	**x**	
Kein gesundheitliches Risiko		**x**

10.1.3 Verhältnis zwischen Bedeutung und Risiko der Studie

Die Untersuchung am Menschen ist erforderlich, da es kein sinnvolles Tiermodell zur Erforschung der gleichen für diese Studie formulierten Fragestellungen gibt und am Tier gewonnene Erkenntnisse besonders in diesem Forschungsbereich nicht auf den Menschen übertragbar sind und daher kaum zum Erkenntnisgewinn einer speziell beim Menschen auftretenden Erkrankung (Harnblasenüberaktivität) beitragen.
Mit dem fMRT steht uns derzeit eine der wenig invasivsten und ungefährlichsten (bei Beachtung der MR-spezifischen Sicherheitsregeln) Untersuchungsmethoden zur Verfügung, um am lebenden Menschen Aussagen über die zerebrale Aktivität unter verschiedenen Untersuchungsbedingungen machen zu können.
Das Verhältnis zwischen Bedeutung und Risiko der Studie erscheint angemessen. Die Erkenntnislücken im Bereich der neuro-urologischen Grundlagenforschung sind gross (siehe Punkt 2.1). Die Patientinnen werden durch die Studie wenig belastet. Schwerwiegende Nebenwirkungen oder unerwünschte Ereignisse sind nicht zu erwarten. Alle Teilnehmerinnen erhalten für den zeitlichen Aufwand eine finanzielle Entschädigung.

11 Qualitätskontrolle und Qualitätssicherung

11.1 Gewährleistung

Diese Studie ist vom Untersucher iniziiert und wird ohne externen Sponsor durchgeführt. Es besteht daher kein externes Monitoring. Ein internes Monitoring wird durch eine nicht in die Studie involvierte Mitarbeiterin, Frau Dr. Dorothee Birnböck durchgeführt. Zugang zu den Originaldaten und Inspektionen durch die Ethikkommission werden gewährleistet. Diese Studie wird nach den Richtlinien der guten klinischen Praxis durchgeführt. Die Qualität der klinischen Betreuung und Behandlung ist durch die Einhaltung der im Managementhandbuch der Uniklinik Balgrist und des UniversitätSpitals festgehaltener Prozesse gewährleistet.
Der Datenpool setzt sich aus verschiedenen Daten zusammen (Tab. 1).

Art der Daten	Form der Daten	Namentliche Zuordnung möglich?	Ort der Aufbewahrung	
Einverständniserklärung	Papierform	Ja	Forschung PZ, Uniklinik Balgrist	
Blasentagebuch	Papierform	Nein	Forschung PZ, Uniklinik Balgrist	
Lebensqualitätfragebogen	Papierform	Nein	Forschung PZ, Uniklinik Balgrist	
CRF	Papierform	Ja	Forschung PZ, Uniklinik Balgrist	
Füllzystometrie	Papierform/ elektronisch	Nein	Forschung PZ, Uniklinik Balgrist	
fMRT	elektronisch	Nein	Forschung PZ, Uniklinik Balgrist / UniversitätsSpital Zürich	
Excel-Datentabelle	elektronisch	Nein	Forschung PZ, Uniklinik Balgrist	

Die Daten von Blasentagebuch (Frequenz, Drang, Inkontinenzepisoden, Flüssigkeitsaufnahme, Verwenden von Einlagen etc.), Lebensqualitätsfragebogen, Einverständniserklärung und CRF (Anamnese, Daten der Füllzystometrie, Resultate der Schwangerschaftstests und Urintests) liegen zunächst nur in Papierform vor. Die Daten der Füllzystometrie liegen zusätzlich auch in elektronischer Form vor und die Daten der fMRT-Messungen liegen ausschliesslich in elektronischer Form vor. Die elektronischen Daten liegen auf sicheren Servern der Universitätsklinik Balgrist (Füllzystometrie) und/oder des UniversitätsSpitals Zürich (fMRT-Daten) und sind bereits in anonymisierter Form (Initialen und fortlaufende Nummerierung, z.B. HM_001 für Hans Meier als erstem Patient) abgespeichert. Eine namentliche Zuordnung der Versuchspersonen zum anonymen Studienkürzel ist nur über den CRF und die Einverständniserklärung im Original möglich.
Auf die Daten in Papierform haben ausschliesslich Mitarbeiter dieser Studie Zugriff. Die elektronischen Daten könnten von anderen, nicht in diese Studien involvierten, Mitarbeitern der Uniklinik Balgrist oder des Universitätsspitals eingesehen werden. Auf Grund der Anonymisierung ist Ihnen jedoch eine Zuordnung der Daten unmöglich. Die Aufschlüsselung der Anonymisierung ist ausschliesslich Mitarbeitern dieser Studie zugänglich.

Alle Daten in Papierform sind in entsprechenden Studienordnern verwahrt (Standort: PZ-Forschung, Uniklinik Balgrist) und werden dann ebenfalls in anonymisierter Form (nur Initialen und fortlaufende Nummer) zur Zusammenfassung und weiteren Auswertung in eine excel-Tabelle übertragen.
Alle Daten werden für mindesten 10 Jahre verwahrt. Der Datenschutz ist während der Studie gewährleistet.

Ort und Datum: ________________________________

Name des Antragstellers: ________________________________

Unterschrift des Antragstellers: ________________________________

Referenzen:

Araki I, Matsui M, Ozawa K, Takeda M, Kuno S. 2003. Relationship of bladder dysfunction to lesion site in multiple sclerosis. The Journal of urology 169(4):1384-1387.

de Seze M, Ruffion A, Denys P, Joseph PA, Perrouin-Verbe B. 2007. The neurogenic bladder in multiple sclerosis: review of the literature and proposal of management guidelines. Multiple sclerosis (Houndmills, Basingstoke, England) 13(7):915-928.

Fowler CJ, Griffiths D, de Groat WC. 2008. The neural control of micturition. Nature reviews 9(6):453-466.

Fowler CJ, Griffiths DJ. 2010. A decade of functional brain imaging applied to bladder control. Neurourology and urodynamics 29(1):49-55.

Griffiths D, Tadic SD. 2008. Bladder control, urgency, and urge incontinence: evidence from functional brain imaging. Neurourology and urodynamics 27(6):466-474.

Griffiths D, Tadic SD, Schaefer W, Resnick NM. 2007. Cerebral control of the bladder in normal and urge-incontinent women. NeuroImage 37(1):1-7.

Heidler S, Deveza C, Temml C, Ponholzer A, Marszalek M, Berger I, Bluhm A, Madersbacher S. 2007. The natural history of lower urinary tract symptoms in females: analysis of a health screening project. European urology 52(6):1744-1750.

Jiang CH, Mazieres L, Lindstrom S. 2002. Cold- and menthol-sensitive C afferents of cat urinary bladder. The Journal of physiology 543(Pt 1):211-220.

Klotz T, Bruggenjurgen B, Burkart M, Resch A. 2007. The economic costs of overactive bladder in Germany. European urology 51(6):1654-1662; discussion 1662-1653.

Mehnert U, Boy S, Svensson J, Michels L, Reitz A, Candia V, Kleiser R, Kollias S, Schurch B. 2008. Brain activation in response to bladder filling and simultaneous stimulation of the dorsal clitoral nerve--an fMRI study in healthy women. NeuroImage 41(3):682-689.

Mehnert U, Michels L, Zempleni MZ, Schurch B, Kollias S. 2010. The supraspinal neural correlate of bladder cold sensation-An fMRI study. Human brain mapping.

Silva C, Ribeiro MJ, Cruz F. 2002. The effect of intravesical resiniferatoxin in patients with idiopathic detrusor instability suggests that involuntary detrusor contractions are triggered by C-fiber input. The Journal of urology 168(2):575-579.

Tadic SD, Griffiths D, Murrin A, Schaefer W, Aizenstein HJ, Resnick NM. 2010a. Brain activity during bladder filling is related to white matter structural changes in older women with urinary incontinence. NeuroImage 51(4):1294-1302.

Tadic SD, Griffiths D, Schaefer W, Cheng CI, Resnick NM. 2010b. Brain activity measured by functional magnetic resonance imaging is related to patient reported urgency urinary incontinence severity. The Journal of urology 183(1):221-228.

Tadic SD, Griffiths D, Schaefer W, Resnick NM. 2008. Abnormal connections in the supraspinal bladder control network in women with urge urinary incontinence. NeuroImage 39(4):1647-1653.

Talairach J, Tournoux P. 1988. Co-planar stereotactic atlas of the human brain. Stuttgart: Thieme.

Wennberg AL, Molander U, Fall M, Edlund C, Peeker R, Milsom I. 2009. A longitudinal population-based survey of urinary incontinence, overactive bladder, and other lower urinary tract symptoms in women. European urology 55(4):783-791.

Zempleni MZ, Michels L, Mehnert U, Schurch B, Kollias S. 2010. Cortical substrate of bladder control in SCI and the effect of peripheral pudendal stimulation. NeuroImage 49(4):2983-2994.
