# Supplementary material for: Urodynamic Investigation: A Valid Tool to Define Normal Lower Urinary Tract Function?
Source: PLoS One. 2016 Oct 13;11(10):e0163847. doi: 10.1371/journal.pone.0163847 (PMC5063299; doi:10.1371/journal.pone.0163847)
Supplement: S4 File — (DOCX) [file pone.0163847.s004.docx]

Translation of the study protocol: Relevant parts

Page 2/21:, Aim:

…Steuerungsvorgänge zur Kontrolle des unteren Harntraktes und deren Veränderung bei Patientinnen mit Dysfunktion des unteren Harntraktes zu erfassen und damit besser zu verstehen…

…to assess the control of the lower urinary tract in healthy and pathological conditions…

Page 6 and 7/21, Design:

Studienablauf

Timetable

… Visite 1 erfolgt eine ausführliche Anamnese und Listung aller ggf eingenommenen Medikamente…. …Die Teilnehmerinnen werden auf ihre Eignung für die Untersuchung untersucht…

…during visit 1 subjects will be examined if they are suitable for the examination

…zudem werden eine urogynäkologische Untersuchung und eine Füllzystometrie (= Blasendruckmessung) zur Sicherung der Diagnose gemäss der Ein- und Ausschlusskriterien durchgeführt.

…further uro-gynecological investigation as well as a filling cystometry (urodynamic investigation), to assess lower urinary tract conditions, and to distinguish if healthy volunteers or patients meet the inclusion criteria, will be performed…

..bei einer Füllzystometrie: wird die Blase mit einer definierten Füllgeschwindigkeit über ein Kathetersystem aufgefüllt und dabei kontinuierlich der intravesikale sowie der näherungsweise über eine rektale Drucksonde gemessene intraabdominale Druck gemessen. Durch Subtraktion des intravesikalen vom intraabdominalen Druck kann somit der Detrusordruck ermittelt werden. Es werden zumindest die Füllmenge und der Detrusordruck zum Zeitpunkt des ersten Harndrangs aufgezeichnet sowie eine Aussage über die maximale Blasenkapazität getroffen. Die Füllzystometrie kann durch den Nachweis isolierter Detrusorkontraktionen oder abnorm hoher Blasendruckwerte im Zusammenhang mit entsprechenden Symptomen der Patientin eine Detrusorüberaktivität belegen bzw. ausschließen. Aus demselben Grund werden die Teilnehmer gebeten, ein Blasentagebuch (s. Anlage) über 3 Tage zu führen und einen Lebensqualitätsfragebogen, der spezifisch für Patienten mit Harnblasenüberaktivität konzipiert und validiert ist, auszufülle...

…filling cystometry consists of bladder filling over an indwelling transurethral catheter; over an implemented pressure sensor intravesical pressure can be measured continuously. A rectal pressure sensor continuously measures the intraabdominal pressure. A subtraction from intravesical and intraabdominal pressure results in the measurement of the detrusor pressure. Abnormal isolated detrusor contractions, maximum detrusor pressures and the bladder capacity can be evaluated. A combination of urodynamic findings in context of patient’s symptoms can lead to the correct diagnosis. Subjects are required to keep a bladder diary for 3 days to address lower urinary tract symptoms in everyday live. Further standardized questionnaires regarding quality of live and lower urinary tract symptoms have to be completed by all subjects…

Page 11/21 Recruitment:

Rekrutierung: Die Rekrutierung der gesunden Probandinnen erfolgt durch Aushang und/oder Annonce…

The recruitment of healthy volunteers is made by public advertisement….

Page 11/21 Inclusion criteria

Gemeinsame Einschlusskriterien für gesunde Probandinnen…

Inclusion criteria for healthy volunteers…

18-55 Jahre; negativer Schwangerschaftstest, schriftliches Einverständnis zur Studienteilnahme

Age between 18 and 55, negative pregnancy test, written informed consent

Page 12/21 Exclusion criteria¨

…Neurologische oder psychologische Erkrankungen, Operationen oder Erkrankungen des Urogenitaltrakte, Fehlbildungen des Urogenitaltraktes, Metabolische Erkrankungen (z.B. Diabetes Mellitus), Symptome einer Harnblasenüberaktivität…

…Neurological or psychological disorders, operations of the lower urinary tract, metabolic diseases (diabetes mellitus), OAB symptoms…

NOTE: In a further amendment male and female participants were both included into the study protocol.

This study was part of a more extensive multimodal project investigating the supraspinal control of the lower urinary tract. Exact details about different protocols (in English) can be found using the included links:

<http://bmjopen.bmj.com/content/4/5/e004357.short>

<http://bmcurol.biomedcentral.com/articles/10.1186/1471-2490-14-68>

The study protocol does include information about all different measurements and sub analyses therefore we only highlighted and translated the important part of this special subproject (supplementary information).
